# Caretta – A multiple protein structure alignment and feature extraction suite

**DOI:** 10.1016/j.csbj.2020.03.011

**Published:** 2020-04-06

**Authors:** Mehmet Akdel, Janani Durairaj, Dick de Ridder, Aalt D.J. van Dijk

**Affiliations:** a Bioinformatics Group, Department of Plant Sciences, Wageningen University and Research, The Netherlands; b Mathematical and Statistical Methods – Biometris, Department of Plant Sciences, Wageningen University and Research, The Netherlands

**Keywords:** Structure alignment, Machine learning, Protein structure, Dynamic programming

## Abstract

The vast number of protein structures currently available opens exciting opportunities for machine learning on proteins, aimed at predicting and understanding functional properties. In particular, in combination with homology modelling, it is now possible to not only use sequence features as input for machine learning, but also structure features. However, in order to do so, robust multiple structure alignments are imperative.

Here we present Caretta, a multiple structure alignment suite meant for homologous but sequentially divergent protein families which consistently returns accurate alignments with a higher coverage than current state-of-the-art tools. Caretta is available as a GUI and command-line application and additionally outputs an aligned structure feature matrix for a given set of input structures, which can readily be used in downstream steps for supervised or unsupervised machine learning. We show Caretta’s performance on two benchmark datasets, and present an example application of Caretta in predicting the conformational state of cyclin-dependent kinases.

## Graphical abstract

**Figure:**
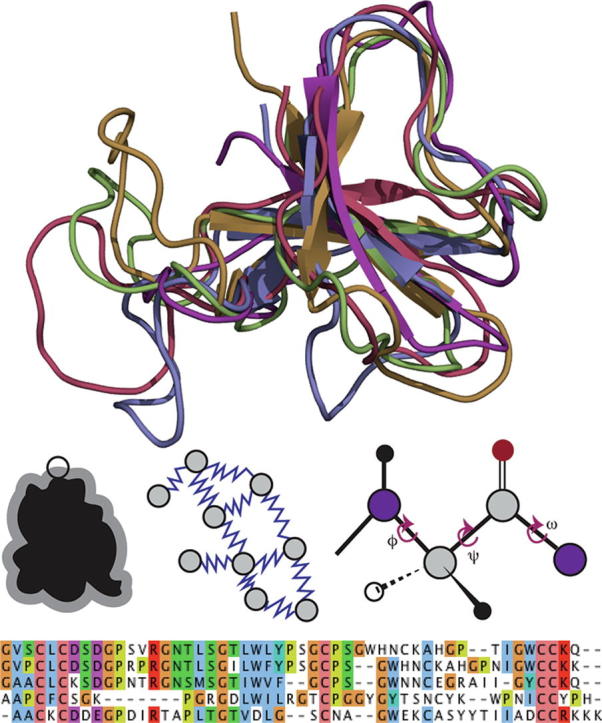

## 1 Introduction

Protein structure alignment has recently been gaining attention in the bioinformatics field, becoming almost as popular as its cousin, protein sequence alignment. While sequence alignment aims to use amino acid substitution patterns and physicochemical properties to make a residue-residue correspondence between sequences of related proteins, structure alignment instead usually focuses on making an optimal superposition of the 3D coordinates of backbone atoms to establish such a correspondence. In many cases these two approaches agree with each other, especially in cases where the proteins under consideration share a high sequence similarity. However, it has been repeatedly observed [14], [16] that some protein families have divergent protein sequences and yet share a high structure, topology, and/or fold similarity, mostly due to the fact that structure tends to evolve slower than sequence [30]. For example, the ubiquitous TIM barrel structural fold is found in over 70 protein families all across nature, and even the most accurate sequence-based techniques cannot find relationships between these diverse sequences with the same structure [27]. In such cases, while sequence alignment may not be successful, structure alignment can still find meaningful residue correspondences.

Until recently, structure alignment has had applications in understanding evolutionary conservation and divergence patterns between proteins across different species [19], identifying conserved active site residues involved in catalytic reactions, creating structure-aware sequence profiles [31], structural similarity search against a database [21] and even as a method to design gold standard datasets for evaluating sequence alignment programs [2], [37]. One area in which comparing multiple protein structures is only recently becoming popular is machine learning.

Though machine learning is not a new field, its popularity and applicability in bioinformatics has recently grown at a tremendous pace. In the protein and enzyme world, machine learning has successfully been applied to predict protein function, protein–protein interactions, drug-target binding, enzyme substrate specificity, thermostability, catalytic rates, binding affinity, and so on [7], [25], [11]. In many of these cases, protein sequences are used due to their widespread availability. However, the increase in both the number of experimentally solved structures, as well as the improvement in structure prediction using homology modeling and co-evolution based approaches, has led to the possibility of incorporating predicted or actual protein structure information (such as residue depth, electrostatic potentials etc.) in machine learning algorithms to better predict and understand outcomes and properties associated with protein families [4], [12].

The typical input for a machine learning algorithm has a tabular format, with each row representing an input protein and each column representing a particular feature or attribute extracted across all the proteins considered. Naturally, the construction of such an input table is often performed by means of a multiple protein alignment. Each column then consists of a particular feature value measured across all the residues in a particular alignment position. This then allows the prediction algorithm to look for patterns in these columns which are correlated with the desired response. For example, in an alignment of ten proteins, if one position is a Trp in the five proteins with a high catalytic rate and a Gly in the five with a lower catalytic rate then this residue position may be implicated in the reduction of catalytic activity. The power of machine learning algorithms lies in finding much more complex and interconnected patterns such as this one. Regions in the alignment with many insertions and deletions, however, can be more difficult to handle, as functionally equivalent residues may be split across multiple columns. This makes it harder for a predictor to spot patterns in a single column or link them together. Often, columns with too many gaps without feature information have to be discarded completely from the analysis, with the risk of losing out on predictive and catalytically important residues simply due to an alignment not fit for the task at hand.

Although there are a number of multiple structure alignment tools, different tools excel in different settings. Many existing multiple structure alignment algorithms, such as Matt [23], [35], MUSTANG [18] and MultiProt [34], focus on and are optimized for aligning evolutionarily distant proteins, which may be from the same superfamily but only share short stretches of structurally conserved “core” regions. Concentrating on these core regions, typically by aligning short fragments of proteins and then assembling these intermediate alignments, leads to these methods overestimating the number of gaps in the alignment, as observed by Carpentier et al. in their multiple structure alignment benchmark [5]. This is especially a hindrance in evolutionarily conserved families as one would expect long stretches of residue correspondences with only a small number of gaps. Therefore, there is a need for a multiple structure alignment tool aimed at returning accurate alignments with a high coverage for homologous protein families with divergent sequences and conserved structures. Machine learning methods which make use of these high-coverage alignments would then have a larger number of extracted residue features at their disposal, allowing for the pinpointing of under-explored residue positions related to an outcome of interest.

Here we present Caretta, a multiple structure alignment tool that additionally outputs aligned structural feature matrices. Caretta uses a combination of dynamic time warping [38] and progressive pairwise alignment [15] to align structures. The pairwise alignment algorithm makes an initial superposition of the two structures using either a signal-based rotation-invariant approach or secondary structure, and further refines the alignment using a scoring system based on the Euclidean distance between corresponding coordinates. The algorithmic novelty of Caretta is that information about the multiple structure alignment is fed into each progressive pairwise alignment in order to maintain and extend existing aligned blocks without disturbing them with insertions unlikely to be found within the same protein family.

We demonstrate that Caretta covers more residues in its alignments than competing tools while still maintaining accuracy. Testing on the widely used Homstrad dataset [26] shows that Caretta often performs on par with manual curation. Caretta is capable of outputting a matrix of features, such as bond angles and residue fluctuations, extracted from the input structures and aligned according to the multiple structure alignment. We use these feature matrices to demonstrate an example workflow of Caretta in machine learning, for classifying cyclin-dependent kinases (CDK) into active or inactive states [22]. Feature selection allows for pinpointing residues involved in state switching, some of which are confirmed by previous studies. A Caretta GUI application allowing for easy access and visualization of aligned structures and features is provided as well. Taken together, Caretta is a full-featured multiple structure alignment suite which provides tools for creating and exploring accurate structural alignments and for calculating structural features extracted from the proteins aligned, in order to successfully apply machine learning to identify distinguishing characteristics of a family of homologous proteins.

## 2 Methods

**Fig. 1:**
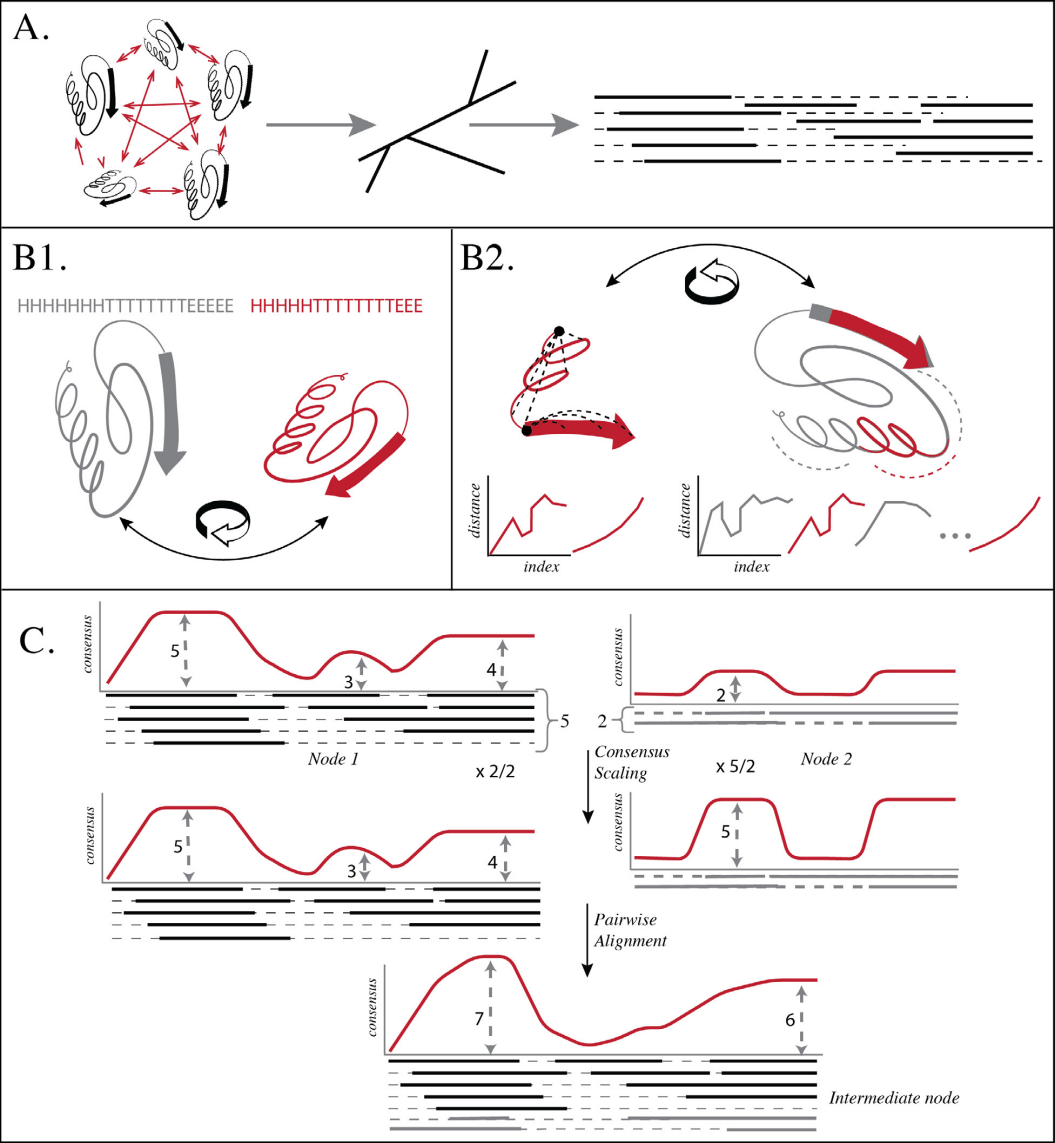
**A.** Caretta’s multiple structure alignment workflow: an all *vs.* all pairwise alignment step, followed by construction of a guide tree and progressive alignment. **B.** The two approaches for initial rotation and superposition of two structures used in pairwise alignment: 1) aligning secondary structure codes and 2) dynamic time warping on one-dimensional signals of distances from all residues to the first or last residue in a segment. **C.** The guide tree specifies the two neighbors to combine at each progressive alignment step. These two neighbors can either be protein structures or previously combined intermediate nodes. A new intermediate node is created at each step by aligning and combining the two neighbors. The alignment step takes into account the number of times each position has been aligned in each of the two neighbors weighted by the number of structures in each neighbor (*consensus* row, shown in red). This ensures that when the difference of the two is taken in calculating ${\mathrm{Score}}_{C}$ (Eq. (3)), positions with fewer gaps get higher scores. After alignment, the *consensus* row of the new intermediate node keeps track of the number of residues aligned at each position.

Fig. 1A depicts the workflow of Caretta for multiple structure alignment: an all *vs.* all pairwise alignment step followed by the construction of a guide tree for progressive alignment, to finally output a multiple alignment. Each intermediate pairwise alignment step uses the dynamic programming approach detailed in Section 2.1. These pairwise alignments use a combination of two different approaches (labelled B1 and B2 in Fig. 1) to construct an initial superposition of structures, described in Section 2.2. The progressive alignment step, explained in Section 2.3 and Fig. 1C, combines aligned structures into an alignment intermediate and boosts the weight of well-aligned residue positions, an approach which reduces the chances of unlikely insertions and deletions.

### 2.1 Dynamic programming based alignment

The algorithm underlying Caretta is dynamic programming alignment with affine gap costs as described by Altschul *et al.* [1]. This algorithm is used in different parts of Caretta with different scoring schemes and different gap open and gap extension penalties, described in the next sections. Supplementary Section 1 contains pseudocode for all the remaining sections with the dynamic programming alignment algorithm represented as *DPAlign*.

### 2.2 Pairwise alignment

Pairwise alignment of two structures depends on the residue-to-residue distance between them. The underlying assumption of a similarity scoring scheme based on residue distance is that the proteins in question are already rotated and centered such that equivalent residues are close to each other. Such a superposition requires a correspondence between residues, *i.e*. an alignment, leading to a chicken and egg problem for pairwise alignment. Caretta solves this by making an initial superposition of two structures using the best out of two coarse alignments: the first based on secondary structure (*SecondarySuperpose* in Supplementary Section 1), and the second based on the alignment of one-dimensional rotation-invariant signals derived from overlapping contiguous segments of the two structures (*SignalSuperpose* in Supplementary Section 1). These two approaches are represented in Fig. 1B1 and Fig. 1B2 respectively, and described below:

1. The first method aligns the residues between two proteins according to their secondary structure elements. The secondary structure score or ${\mathrm{Score}}_{S}$ is defined as below, where $s_{i}$ represents the DSSP secondary structure code (Supplementary Table 1) for residue *i*:
  (1) $${\mathrm{Score}}_{S}(i,j)=\left\{\begin{array}{c} \;\;\;0\;\mathrm{if}\;s_{i}=‘-’\;\vee \;s_{j}=‘-’\\ \;\;\;1\;\mathrm{if}\;s_{i}=s_{j}\\ \;\;\;\;\;\;\;-1\;\mathrm{if}\;s_{i}\;\neq \;s_{j} \end{array}\right)$$
  This scoring system is used with gap open and gap extend penalties $\sigma_{S}=1$ and ${∊}_{S}=0$ (since this scoring scheme works in increments or decrements of 1) to make an initial alignment. The two proteins are then superposed using the Kabsch algorithm [17] to find the rotation and translation matrix that optimally matches the aligning pairs of residues.
2. The second method performs dynamic time warping on rotation-invariant overlapping segments of two structures. Each segment represents each residue *r* in a thirty-residue stretch of the structure by the Euclidean distances of its α-carbon to the α-carbon of the first residue in the segment ($\overset{\to }{P}=\left[d_{0},d_{1},\ldots ,d_{n}\right]$). The score between two such segments is given as:
  (2) $${\mathrm{Score}}_{P}(i,j)={\mathrm{median}}_{d}\left(\exp\left(-\frac{{\left({\overset{\to }{P}}_{i,d}-{\overset{\to }{P}}_{j,d}\right)}^{2}}{10}\right)\right)$$
  After determining the alignment of these segments (by using ${\mathrm{Score}}_{p}$ with zero gap penalties to allow for more leniency as the proteins are not yet in their correct orientation), the optimal rotation and translation of the α-carbons of the first residues in each aligning pair of segments are calculated using the Kabsch algorithm [17] and used to superpose the two structures.
  This approach is repeated, taking the distances to the last α-carbon in each segment instead of the first, to obtain a different superposition.

The superposition from the above two approaches giving the best-scoring alignment is chosen. The scoring method used by Caretta uses an RBF (Gaussian) kernel derived from the Euclidean distance between two (superposed) α-carbon coordinates ($\overset{\to }{\alpha }=\left[\alpha_{x},\alpha_{y},\alpha_{z}\right]$), defined below:

(3) $${\mathrm{Score}}_{C}(i,j)=\exp\left(-\gamma \sum {\left(\overset{\to }{\alpha_{i}}-\overset{\to }{\alpha_{j}}\right)}^{2}\right)$$

Supplementary Fig. 2 shows the distribution of this score for different values of γ as a function of Euclidean distance. We chose a γ value of 0.03 as this causes a sharp drop to near-zero values at 8 Å while still yielding a score of around 0.6 at the commonly used structural equivalence cutoff of 4 Å, reflecting the belief that residues further away than 8 Å are not likely to be structurally or functionally equivalent.

This score is summed across all paired residues to derive the score of an alignment between two proteins *x* and *y*:

(4) $${\mathrm{PairwiseAlignmentScore}}_{C}(x,y)=\underset{(i,j)\in \mathrm{aligned}\;\mathrm{residue}\;\mathrm{pairs}}{\sum }{\mathrm{Score}}_{C}(x_{i},y_{i})$$

Caretta uses the scoring scheme ${\mathrm{Score}}_{C}$ and $\sigma_{C}$ and ${∊}_{C}$ as gap open and gap extend penalties (set to 1 and 0.01 for the alignments presented here), on the newly superposed coordinates to find the optimal correspondence between them (*PairwiseAlignment* in Supplementary Section 1).

When more than two structures are required to be aligned, pairwise alignments are made for all input structures. This step is essential for the guide tree construction described in the next section.

### 2.3 Multiple alignment

The idea behind a progressive alignment approach is to perform step-wise alignments of two stacks of previously aligned structures (or single structures) to result in a final stack of all aligned structures. The order of addition of structures is a crucial factor in the performance of this method. Aligning similar structures first, with a smoother progression towards distantly related structures, increases the chances of a good alignment. We construct a guide tree for determining the order of progression using maximum linkage neighbor joining [32] on the pairwise alignments constructed in Section 2.2. The pairwise tree score for two proteins is given by their pairwise alignment score (Eq. (4)) divided by the number of aligning pairs.

With the guide tree in place, the progressive alignment steps start, as illustrated in Fig. 1C and Supplementary Section 1 *MultipleAlignment*. While progressive alignment typically consists of independent pairwise alignment steps, the algorithmic novelty of Caretta lies in the introduction of a feedback loop between the state of the multiple structure alignment and each pairwise alignment, explained in detail below. For this purpose, an additional *consensus* row, of length equal to the protein length, is maintained for each structure, initiated with a consensus weight parameter (*cw*, default = 1). This row is concatenated to the coordinates $\overset{\to }{\alpha }$ of a protein before ${\mathrm{Score}}_{C}$ in Eq. (3) is calculated.

Before two neighbors in the guide tree are aligned, the *consensus* row of each neighbor is multiplied by half the number of structures represented by the other neighbor. This ensures that when their difference is taken during the calculation of ${\mathrm{Score}}_{C}$ in Eq. (3) (with the *consensus* row attached), the positions with equal *consensus* values in both receive high scores (as the difference is close to zero), increasing their chances of being aligned. An intermediate node is created with a length equal to the length of the resulting pairwise alignment, representing the aligned stack of structures from the two neighbors. The x,y, and *z* coordinates of this intermediate node are calculated by averaging the coordinates of the two initial structures after superposition, across the alignment. At each position in the alignment, the secondary structure code in the intermediate node is taken as the code of the input which does not have a gap, or the code of the first input if both are aligned. The *consensus* term for each alignment position is the number of aligned residues at that position times the *cw*, i.e. well-aligned positions with fewer gaps have a higher *consensus* value. This way, Caretta tends to maintain fully aligned core regions by avoiding the insertion of gaps at these locations as progressive alignment proceeds as such gaps are unlikely to happen in conserved protein families.

### 2.4 Benchmarking

#### 2.4.1 Data

Caretta takes as input a list of PDB files, along with optional chain identifiers and start and end residue indices. All PDB file parsing is done using ProDy [3] and the secondary structure for each protein is derived using ProDy’s execDSSP [36] function.

Caretta was tested on two benchmark datasets, Homstrad [26] and SABmark-Sup [37]. The PDB files for these two datasets were obtained from mTM-align’s website [9] and Matt benchmark results [23] [24] respectively, in order to directly compare results to the output of these two tools. To this end, the alignments for the Homstrad [26] and SABmark-Sup [37] datasets for Matt [23] and mTM-align [8] were obtained from Mattbench [24] and mTM-align’s website [9] respectively. For 35 cases in the SABmark-Sup dataset, mTM-align returned alignments where at least one sequence did not match the corresponding PDB sequence. These cases are not shown in Fig. 4A. Two protein families, labelled seatoxin and kringle in the Homstrad benchmark set are used to demonstrate and contrast the alignments returned by Caretta, Matt and mTM-align. The structures in these groups are superposed according to the gap-less positions in each alignment and visualized using Pymol [33].

#### 2.4.2 Metrics

To measure the quality of multiple structure alignments we make use of various metrics. The last two are defined for pairwise alignments, and are calculated for every pair of structures in the multiple structure alignment after superposing all structures to one reference structure, the longest protein. These are then averaged over all pairs to give the final score for a multiple alignment.

• **Gap-less positions** – Positions in an alignment that do not contain any gaps.
• **Homstrad equivalence score** – Percentage of gap-less positions which are present in the corresponding Homstrad reference alignment.
• **RMSD** – The root mean square deviation between two superposed structures in a pairwise alignment is given by:
  $$\sqrt{\frac{\sum_{(i,j)\in \mathrm{aligned}\;\mathrm{residue}\;\mathrm{pairs}}{\left(\overset{\to }{\alpha_{i}}-\overset{\to }{\alpha_{j}}\right)}^{2}}{\left|\mathrm{aligned}\;\mathrm{residue}\;\mathrm{pairs}\right|}}$$
• **Structurally equivalent residues** – Residues in the same alignment position of a pairwise alignment within 4 Å of each other after superposition.

#### 2.4.3 Measuring runtime

To estimate Caretta running times, we randomly chose 25 protein structures from the Homstrad dataset with differing lengths as “seeds”. Each seed was used to form multiple groups of proteins to be aligned by Caretta. Forming each of these groups involved introducing noise to the seed coordinates to create a given number of members, from 13 to 93 in increments of 30. Caretta was then used to align these groups on a Linux workstation using 20 threads.

### 2.5 Feature extraction

In addition to multiple structure alignment, Caretta was designed specifically in order to enable feature extraction for downstream machine learning.

Structural features are extracted for each input protein, aligned according to Caretta’s multiple structure alignment. All atom-level features are converted into α-carbon, β-carbon, and mean residue features. For Gly, the α-carbon is used for the β-carbon features as well.

ProDy [3] is used to calculate the 50-mode Gaussian Network Model (GNM) and Anisotropic Network Model (ANM) atom fluctuations using the calcGNM/calcANM functions followed by the calcSqFlucts function.

DSSP features are calculated using ProDy [3] to give hydrogen bond energies, surface accessibility, dihedral angles (α), bend angles (κ), ϕ, and ψ backbone torsion angles, and tco angles (cosine angle between the C = O of residue *i* and the C = O of residue i-1).

Residue depths are extracted using BioPython [6].

### 2.6 Cyclin-dependent kinase classification

Caretta’s alignment and feature extraction capabilities are further demonstrated on the task of predicting the functional state of cyclin-dependent kinases (CDKs). PDB IDs of these proteins, along with the corresponding active/inactive labels, were obtained from [22]. These proteins were clustered by sequence similarity using an LZW kernel [13], and a single cluster containing 80 CDKs was chosen. A multiple structure alignment was made for these 80 CDKs followed by feature extraction of DSSP features, GNM and ANM fluctuations and residue depths. These features were aligned after discarding positions in the alignment which contained gaps. A logistic regression model with L1 penalty was trained for binary classification of CDK active/inactive state, and tested on 50 random splits of the data, each with 60 training points and 20 test points, using the scikit-learn Python library [28]. The importance of an alignment position was taken to be the sum of the absolute values of the feature coefficients for that position, averaged across all train/test splits. This scoring scheme was used to select the top 15 most informative residue positions, which were visualized on the proline-rich tyrosine kinase 2 (PYK2) CDK structure (PDB ID:3FZP, chain A) using PyMOL [33].

### 2.7 GUI application

The Caretta GUI was built using Dash and Dash-Bio [29]. It takes as input a list of PDB IDs, either from a user-specified folder or from a list of structures associated with a user-inputted Pfam domain, and performs multiple structure alignment on these structures. The results are displayed in three different panels:

• Structure alignment – displays the superposed 3D structures of the input proteins;
• Sequence alignment – displays the multiple sequence alignment, colored by hydrophobicity;
• Feature alignment – displays aligned structural features. The feature name under consideration can be changed using a drop-down box.

These three panels are interlinked via interactive capabilities. Clicking a protein or a residue position in any of the three panels highlights the corresponding protein or position in the other two. All three panels can also be exported to different file formats for downstream use.

## 3 Results

### 3.1 Caretta returns accurate alignments with higher coverage

We compare Caretta with two popular multiple structure alignment methods, Matt and mTM-align. Matt uses a fragment-based approach, which allows for local flexibility between fragment pairs from two input structures and then a dynamic programming algorithm to assemble these intermediate pairs [23]. mTM-align [8] instead performs global alignment and builds upon the pairwise structure alignment algorithm TM-align [39], which uses the length-independent TM-score as a measure of similarity between two proteins in a dynamic programming approach. mTM-align then progressively assembles these pairwise alignments into a multiple structure alignment.

These two MSA tools were tested along with Caretta on the popular Homstrad and SABmark-Sup datasets. Assessing the quality of multiple structure alignments is a difficult task and, depending on the metric used, different aspects of the alignment come under consideration. While RMSD (root mean square deviation) is often used, it has been observed that fewer aligned residues can easily lead to smaller RMSDs at the expense of a very gap-filled alignment [8], which can easily happen in the case of proteins with conserved cores but flexible regions that are not often aligned. While the conserved core can be responsible for the overall stability and function of the protein, the flexible regions can occur in and around active sites or interaction sites and lead to differences in enzyme specificity towards substrates, products, or interaction partners [20] – making them immensely important for machine learning aimed at predicting determinants of such specificities. Thus, gap-filled alignments focusing on low RMSDs, while accurate and useful for superposition of structures, are sub-optimal for machine learning as the features of many potentially relevant residues are discarded due to a lack of data in those positions. In most cases, positions with over a certain percentage of aligned residues are considered, with gaps replaced by zeros or by the average of the feature values in that position [22]. Therefore, when benchmarking Caretta we emphasize the coverage of the alignment along with structural equivalence measures such as RMSD.

**Fig. 2:**
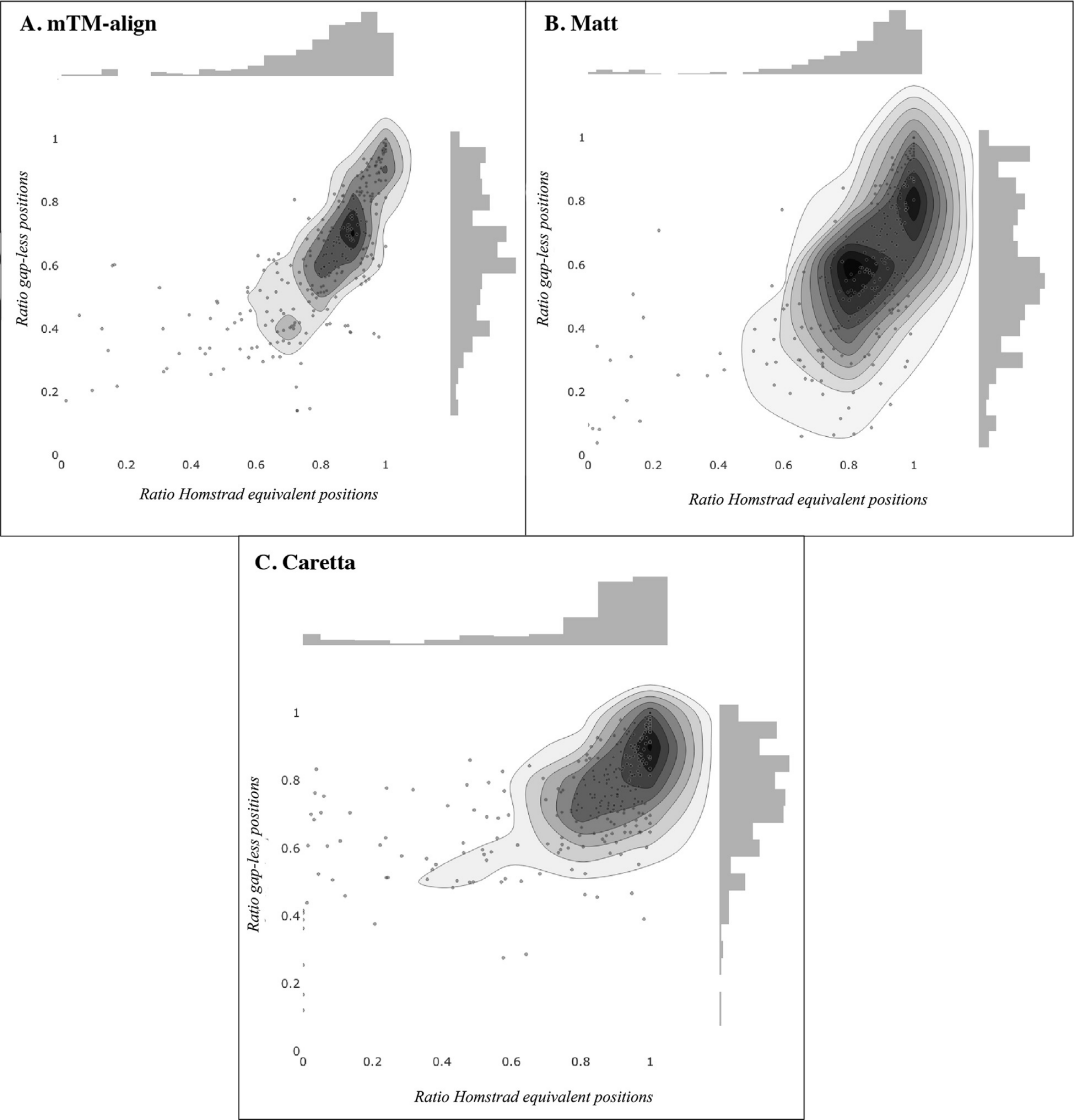
Plots showing the ratio of gap-less positions in an alignment which are identical to the corresponding Homstrad reference alignment vs. the ratio of all gap-less positions. **A**, **B**, and **C** show the results for alignments generated by mTM-align, Matt, and Caretta respectively.

The Homstrad dataset is unique in that it provides manually curated and annotated alignments, representing a ground truth. This dataset has examples from various homologous protein families, typical of the kinds of applications where machine learning would be applied. Since these proteins are homologous, a high alignment coverage is expected as many of the residues are functionally equivalent, with few insertions and deletions. In Fig. 2 we show the percentage of gap-less columns found by each aligner that are the same as the corresponding column in the Homstrad reference (Homstrad equivalence score), against the percentage of all gap-less columns in the alignment. Caretta clearly outperforms the other aligners by regularly finding near-optimal alignments with a high coverage. In the majority of cases (65% for Matt, and 82% for mTM-align), Caretta also finds the same or more structurally equivalent residues within gap-less positions. Taken together, this indicates that the increase in gap-less positions is warranted in that Caretta still finds accurate residue pairings.

**Fig. 3:**
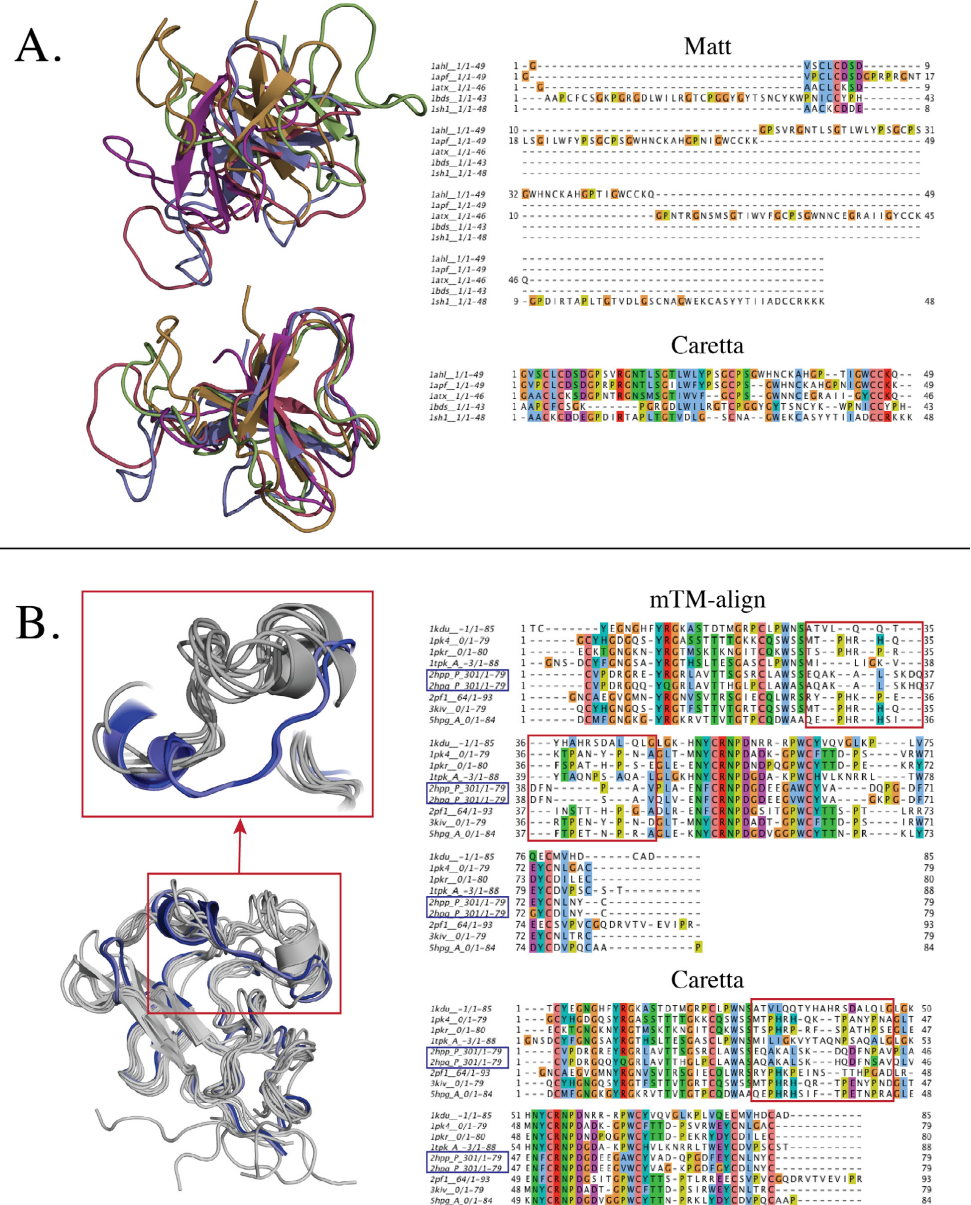
Two examples of protein families in which Caretta finds a better alignment than Matt and mTM-align. **A.** structures from the “seatoxin” family (a collection of toxins released by sea anemones), superposed according to alignments made by Matt (top) and Caretta (bottom) respectively, with the alignments shown on the right. **B.** structures from the “Kringle” family (PFAM ID: PF00051) superposed according to Caretta’s alignment. Two structurally divergent proteins are highlighted in blue and the region of divergence is highlighted in red, both in the structure superposition and in the corresponding alignments on the right. These two groups of proteins are obtained from the Homstrad benchmark dataset [26].

Fig. 3 shows two examples where Caretta does a better job of multiple structure alignment in terms of Homstrad equivalence. The first case shows a family of small, loop-filled structures where the pitfalls of optimizing for RMSD become clear. Matt, in this case, only gap-lessly aligns 8 residues and has a Homstrad equivalence of 3%, while Caretta achieves a Homstrad equivalence score of 58% by correctly aligning areas where structural flexibility makes it difficult to accurately pinpoint equivalent residue pairs. The second case demonstrates a family in which some members structurally deviate from the others in a small region. Such regions are especially relevant for machine learning as they may be responsible for a change in a response variable such as substrate specificity, catalytic rate etc. Both Caretta and mTM-align lead to the same superposition of structures for this family but mTM-align inserts gaps in the highlighted region such that the two divergent proteins cannot be compared here.

**Fig. 4:**
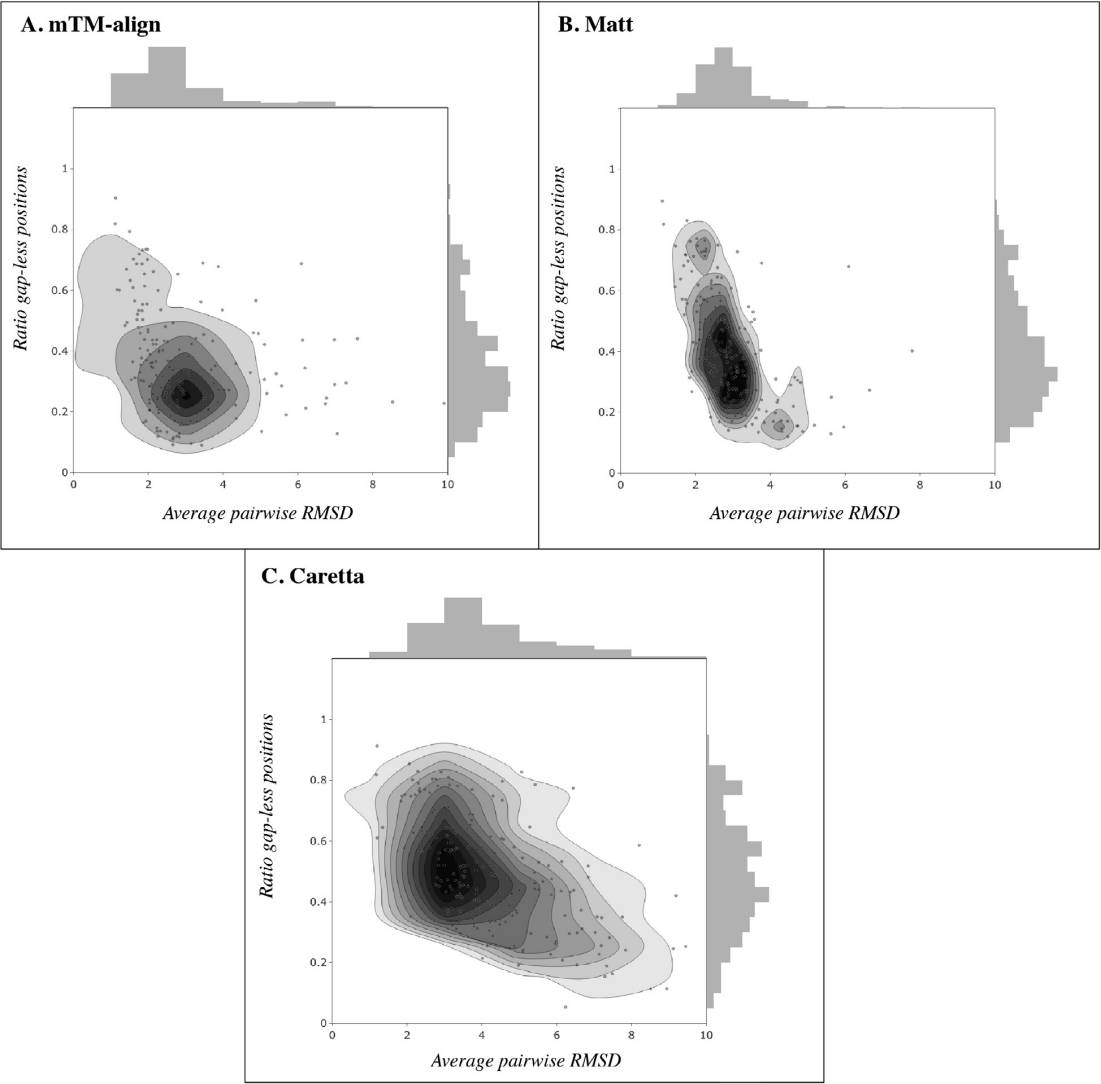
Plots showing average pairwise RMSD vs. ratio of gap-less alignment positions across the alignments in the SABmark-sup dataset. **A**, **B**, and **C** show the results for alignments generated by mTM-align, Matt, and Caretta respectively.

The SABmark-Sup benchmark dataset consists of proteins from the same superfamily with distant homology [37]. These proteins are much harder to align as usually only small fragments have any meaningful correspondence. Though Caretta has adjustable gap penalties that can be useful in such cases to allow for substructure alignment, these are still not the optimal conditions for the algorithm. While Matt is known to yield alignments with low RMSDs in this dataset, this is at the expense of coverage, which is often quite low. Fig. 4 shows the average RMSD *vs.* average percentage of gap-less columns for Matt, mTM-align and Caretta on the SABmark-Sup dataset. While Matt and mTM-align have a gap-less percentage range typically within 20–60%, this increases to 40–80% for Caretta, often still within the same RMSD range. This indicates that Caretta also performs well at fragmented substructure alignment, though optimizing the gap penalties and consensus weight may improve results further for individual cases.

**Fig. 5:**
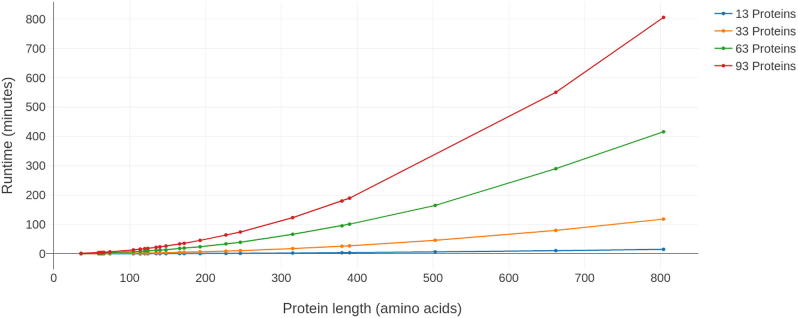
Runtime measured in minutes for Caretta alignment using 20 threads on proteins of differing lengths (constructed as described in Section 2.4.3. Each line represents a different number of proteins aligned.

The time complexity of Caretta’s alignment algorithm is $O(n^{2}l^{2})$ where *n* is the number of proteins and *l* is the length of the longest protein in the alignment. Fig. 5 shows the time taken for Caretta alignment for varying numbers and lengths of proteins. These results show that aligning a reasonably large set of protein structures (50–90) with a mid-range residue length (200–300) takes less than 2 h on a workstation with 20 threads.

### 3.2 An application of Caretta in predicting Cyclin-dependent kinase conformation

Apart from α-carbon coordinates, residues in a protein carry a wealth of structural information, as a result of the physicochemical differences between amino acids and the many interactions to neighboring residues. This information can be extracted from structures and used to explore differences and similarities between proteins in the same family performing different functions. To enable such exploration, Caretta calculates and outputs various structural features aligned according to the core columns in the multiple structure alignment. The feature matrices outputted by Caretta can be also used for downstream tasks such as dimensionality reduction and supervised learning. As proteins typically have many hundreds of residues each with tens of features, a feature selection step is recommended for small datasets, to focus on functionally important residues.

An application of Caretta for a classification task is presented using a dataset of cyclin-dependent kinases (CDKs). This family of enzymes is involved in cell cycle regulation and its members share a high degree of structural similarity. Classical kinase inhibitors bind to the ATP site of CDKs and compete for substrate binding [10]. The determination of additional inhibitor binding sites in these enzymes, which would switch their state from active to inactive in terms of substrate binding, is a challenging and significant problem in the drug design field. One intriguing aspect of this family is that the same or very similar sequences can switch state depending on their structural conformation which means that sequence similarity cannot be successfully used for classification [22].

**Fig. 6:**
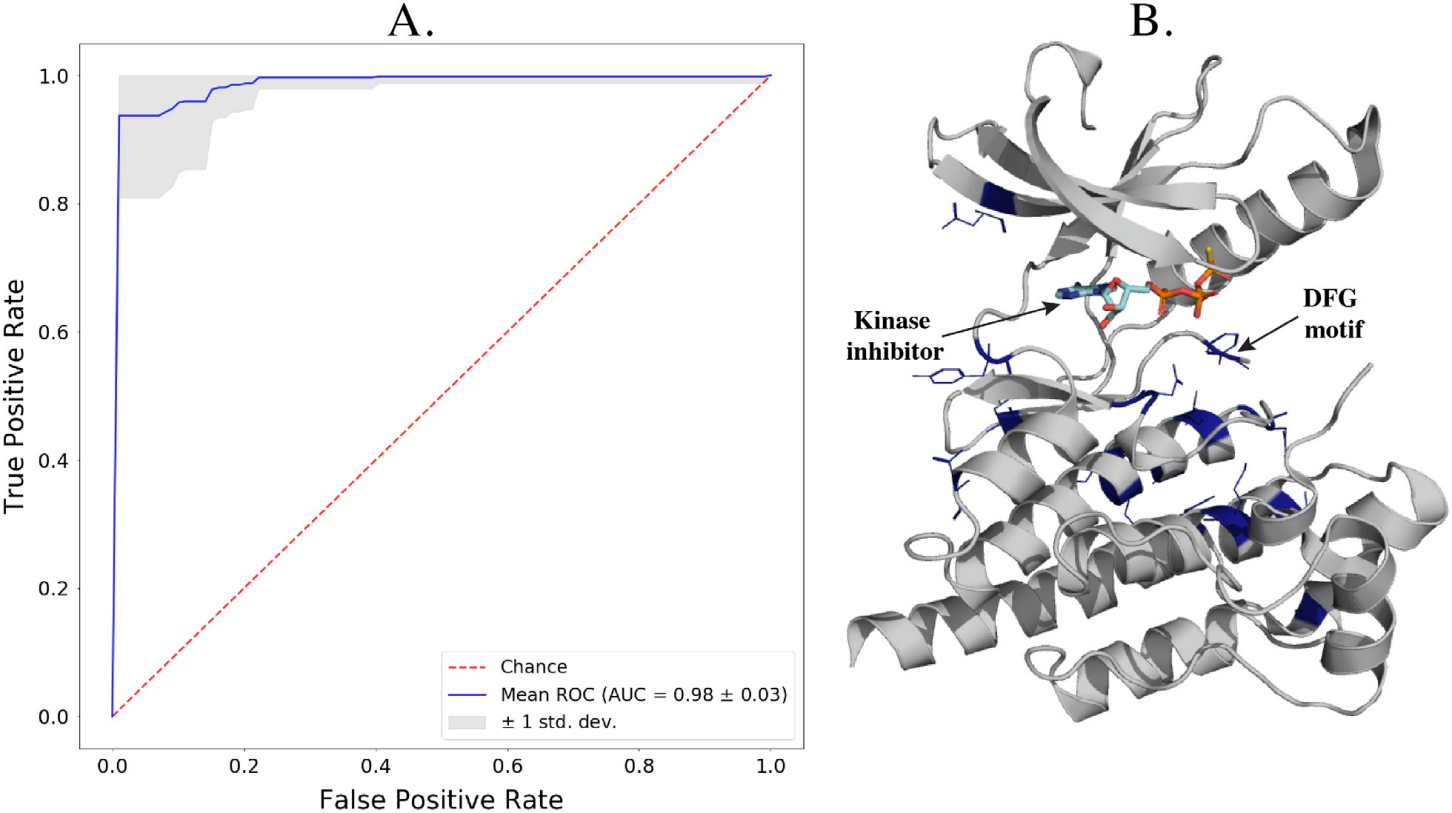
**A.** ROC-AUC curve showing the cross-validation performance of the logistic regression model to predict the state (active/inactive) of cyclin-dependent kinases (CDKs). This model is trained on structural residue features (bond angles, residue depths, fluctuations, and solvent accessibilities), and aligned according to Caretta’s multiple structure alignment of the CDKs. **B.** Structure of active proline-rich tyrosine kinase 2 (PYK2), co-crystallized with the ATPγS ATP-mimetic kinase inhibitor. The catalytically important DFG motif is labelled and the fifteen most predictive residues for active/inactive state determination are colored in blue and represented as sticks.

Fortunately, a large number of CDK structures have been experimentally solved. We used Caretta to align 80 CDK structures and extracted bond angles, Gaussian and anisotropic network model residue fluctuations, residue depths and solvent accessibility features from these structures. The alignment had a mean pairwise RMSD of 3.08 Å and 128 gap-less positions. We trained a logistic regression model to predict active/inactive states of CDKs using features aligned according to these gap-less positions and attained a mean cross-validation accuracy of 98%, with only 60 structures used for training in each split. The performance is summarized in Fig. 6A in a Receiver operating characteristic (ROC) curve. Summing the absolute values of the feature coefficients for a residue across all splits allowed us to rank informative residue positions and pinpoint residues relevant to the activation process, as shown in Supplementary Fig. 3. Fig. 6B labels the fifteen most informative residues on the structure of inactive proline-rich tyrosine kinase 2 (PYK2), co-crystallized along with an ATP-mimetic kinase inhibitor (ATPγS). Supplementary Fig. 4 shows a PCA plot of the feature values of these top 15 residues, demonstrating a clear distinction between active and inactive CDKs. Interestingly, a number of the selected residues lie close to the inhibitor, with one falling within the well-studied DFG motif [10], indicated in the figure. The remaining selected residues cluster underneath this motif, indicating flexibility in these regions associated with a conformational change. This simple example demonstrates the power of a robust structural alignment, combined with features describing various aspects of protein structures, in exploring distinguishing characteristics of protein families. Insights gained from such studies can be utilized for mutational studies to engineer enzymes with desired activity, or in inhibitor design. While CDKs are relatively unique in that there are many solved crystal structures, due to the advances in homology modelling as well as the growing size of the PDB, most protein families can be supplemented with accurate structural models, which can then be aligned and analysed in a similar way with Caretta.

### 3.3 Caretta can be used to visually explore structure alignments and features.

**Fig. 7:**
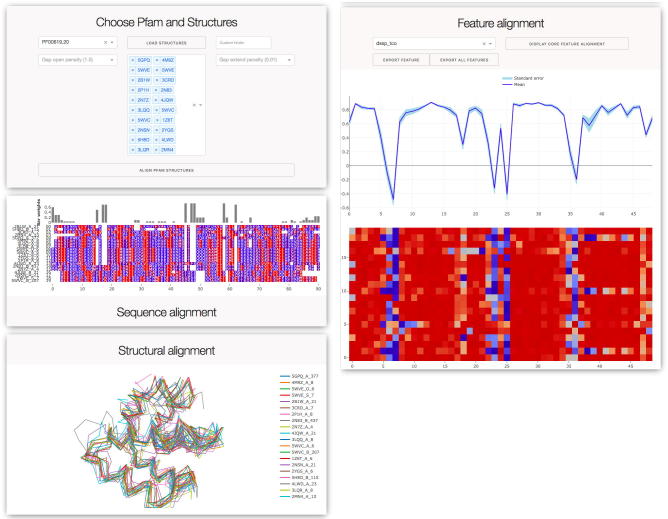
An example of Pfam domain alignment possible with the Caretta website (found at www.bioinformatics.nl/caretta). The user selects a Pfam domain and is given the list of PDB IDs associated with that domain. The website allows selection of up to 40 PDB IDs to align. Once alignment is complete, three panels are displayed, showing the multiple sequence alignment, the corresponding superposition of the structures, and the alignment of structural features (with a drop-down menu to choose between different features). These three panels are interconnected, allowing the user to select proteins and residue positions across all three views at once.

A Caretta GUI application can be found at www.bioinformatics.nl/caretta for aligning selected structures, from either a Pfam domain or a custom folder, and exploring their structural features. Fig. 7 shows the kind of information that can be obtained. The application is fully interactive, with the sequence and feature alignments linked to the corresponding residues in the structure alignment. Different features such as bond and torsion angles, electrostatics, atom fluctuations etc. can be visualized separately, and the means and standard deviations across all proteins are shown for each position allowing the user to easily pinpoint highly variable or highly conserved residues or residues. While the website only allows for the alignment of up to 40 structures, the application can be installed locally to avoid this restriction. In addition, Caretta can also be installed as a command line application or used as a Python library for easy handling of multiple structures, features, and alignments.

## 4 Conclusion

Multiple sequence alignment is an integral part of a broad range of bioinformatics research topics, including phylogenetics, functional domain identification, co-evolution analysis and machine learning to predict functional properties of proteins. Compared to protein sequences, protein structures echo an even deeper evolutionary history that in a more direct way relates to their function. Previously, this kind of analysis was hindered by the scarcity of protein structures available. However, the number of solved protein structures is increasing at a great pace, and structural modelling methods are also improving rapidly, in part due to the use of co-evolutionary information when reliable structural templates are not available. This means it is now possible to analyse patterns correlating with function in a protein family by aligning, comparing and applying machine learning on a large set of solved or modeled structures.

We contribute to this field with Caretta, a multiple structure alignment suite which returns accurate alignments with an increased ratio of aligned positions to make the best use of structural features from functionally comparable residues. Dong et al. [8] noticed that the accuracy of a multiple structure alignment depends heavily on the quality of the individual pairwise alignments, which in turn depends on the initial superposition of two proteins, often accomplished by approximate point cloud registration techniques. Caretta uses signals of distances derived from overlapping contiguous stretches of residues to make this initial superposition, a novel rotation-invariant technique. This, combined with a novel feedback approach to maintain well-aligned blocks of residues in the multiple alignment, works well with protein families where large and numerous stretches of insertions are not expected to be found.

In the Caretta GUI, we coupled structural alignment and feature extraction with a visual interface to pinpoint relevant proteins and residue positions for downstream prediction tasks. This kind of feature selection becomes necessary as proteins typically have many hundreds of residues, each of which is described by a number of structural features. This quickly leads to what is known as the “large **p** small **n**” problem in machine learning, where the number of descriptors far exceeds the number of labeled data points from which to learn. Feature selection in such cases removes noisy and irrelevant features, and can be used to find residue positions correlated with the response variable. We demonstrated this in our application on predicting the conformational state of cyclin-dependent kinases, where we found a small set of predictive residues, some of which lie in previously studied motifs known to be involved in conformational change.

More research into protein families using the approach we present for dealing with structural alignments and residue selection across a large set of structural features will lead to improvements and novel techniques for feature selection, dimensionality reduction, and learning that work well on such large, hierarchically structured data. Given the prominent role in present-day bioinformatics of both machine learning and homology modelling, this will lead to further breakthroughs in using protein structures to analyse protein function.

## Conflicts of interest

The authors declare no conflicts of interest.

## CRediT authorship contribution statement

**Mehmet Akdel:** Conceptualization, Methodology, Software, Validation, Formal analysis, Writing - original draft. **Janani Durairaj:** Conceptualization, Methodology, Software, Validation, Formal analysis, Writing - original draft. **Dick de Ridder:** Writing - review & editing, Supervision. **Aalt D.J. van Dijk:** Writing - review & editing, Supervision, Project administration.
