# Supplementary material for: Caretta – A multiple protein structure alignment and feature extraction suite
Source: Comput Struct Biotechnol J. 2020 Apr 6;18:981–92. doi: 10.1016/j.csbj.2020.03.011 (PMC7186369; doi:10.1016/j.csbj.2020.03.011)
Supplement: Supplementary file 1 [file mmc1.zip › doc/elsdoc.pdf]

elsarticle.cls – A better way to format your document

Elsevier Ltd

QUICK LINKS

- ▶ Introduction
- ▶ Installation
- ▶ Frontmatter
- ▶ Theorem and ...
- ▶ Cross-references
- ▶ Bibliography
- ▶ Final print
- ▶ Major Differences
- ▶ Usage
- ▶ Floats
- ▶ Enumerated ...
- ▶ Mathematical ...
- ▶ Graphical ...

1. Introduction

elsarticle.cls is a thoroughly re-written document class for formatting LaTeX submissions to Elsevier journals. The class uses the environments and commands defined in LaTeX kernel without any change in the signature so that clashes with other contributed LaTeX packages such as hyperref.sty, preview-latex.sty, etc., will be minimal. elsarticle.cls is primarily built upon the default article.cls. This class depends on the following packages for its proper functioning:

1. natbib.sty for citation processing;
2. geometry.sty for margin settings;
3. fleqn.clo for left aligned equations;
4. graphicx.sty for graphics inclusion;
5. txfonts.sty optional font package, if the document is to be formatted with Times and compatible math fonts;
6. hyperref.sty optional packages if hyperlinking is required in the document;
7. endfloat.sty optional packages if floats to be placed at end of the PDF.

All the above packages (except some optional packages) are part of any standard LaTeX installation. Therefore, the users need not be bothered about downloading any extra packages. Furthermore, users are free to make use of AMS math packages such as amsmath.sty, amsthm.sty, amssymb.sty, amsfonts.sty, etc., if they want to. All these packages work in tandem with elsarticle.cls without any problems.

2. Major Differences

Following are the major differences between elsarticle.cls and its predecessor package, elsart.cls:

- elsarticle.cls is built upon article.cls while elsart.cls is not. elsart.cls redefines many of the commands in the LaTeX classes/kernel, which can possibly cause surprising clashes with other contributed LaTeX packages;
- provides preprint document formatting by default, and optionally formats the document as per the final style of models 1+, 3+ and 5+ of Elsevier journals;

© 2019, Elsevier Ltd. Bugs, feature requests, suggestions and comments shall be mailed to <elsarticle@stmdocs.in>.

Version: 3.2
Date: April 5, 2019
Contact: elsarticle@stmdocs.in

- some easier ways for formatting `list` and `theorem` environments are provided while people can still use amsthm.sty package;
- natbib.sty is the main citation processing package which can comprehensively handle all kinds of citations and works perfectly with hyperref.sty in combination with hypernat.sty;
- long title pages are processed correctly in preprint and final formats.

3. Installation

The package is available at author resources page at Elsevier (http://www.elsevier.com/locate/latex). It can also be found in any of the nodes of the Comprehensive TEX Archive Network (CTAN), one of the primary nodes being http://tug.ctan.org/tex-archive/macros/latex/contrib/elsarticle/. Please download the elsarticle.dtx which is a composite class with documentation and elsarticle.ins which is the LATEX installer file. When we compile the elsarticle.ins with LATEX it provides the class file, elsarticle.cls by stripping off all the documentation from the `*.dtx` file. The class may be moved or copied to a place, usually, `$TEXMF/tex/latex/elsevier/`, or a folder which will be read by LATEX during document compilation. The TEX file database needs updation after moving/copying class file. Usually, we use commands like `mktexlsr` or `texhash` depending upon the distribution and operating system.

4. Usage

The class should be loaded with the command:

```
\documentclass[<options>]{elsarticle}
```

where the `options` can be the following:

`preprint` default option which format the document for submission to Elsevier journals.

`review` similar to the `preprint` option, but increases the baselineskip to facilitate easier review process.

`1p` formats the article to the look and feel of the final format of model 1+ journals. This is always single column style.

`3p` formats the article to the look and feel of the final format of model 3+ journals. If the journal is a two column model, use `twocolumn` option in combination.

`5p` formats for model 5+ journals. This is always of two column style.

`authoryear` author-year citation style of natbib.sty. If you want to add extra options of natbib.sty, you may use the options as comma delimited strings as arguments to `\biboptions` command. An example would be:

```
\biboptions{longnamesfirst,angle,semicolon}
```

`number` numbered citation style. Extra options can be loaded with `\biboptions` command.

`sort&compress` sorts and compresses the numbered citations. For example, citation [1,2,3] will become [1–3].

`longtitle` if front matter is unusually long, use this option to split the title page across pages with the correct placement of title and author footnotes in the first page.

`times` loads txfonts.sty, if available in the system to use Times and compatible math fonts.

`reversenotenum` Use alphabets as author–affiliation linking labels and use numbers for author footnotes. By default, numbers will be used as author–affiliation linking labels and alphabets for author footnotes.

`lefttitle` To move title and author/affiliation block to flushleft. `centertitle` is the default option which produces center alignment.

`endfloat` To place all floats at the end of the document.

`nonatbib` To unload natbib.sty.

`doubleblind` To hide author name, affiliation, email address etc. for double blind refereeing purpose.

All options of article.cls can be used with this document class.

The default options loaded are `a4paper`, `10pt`, `oneside`, `onecolumn` and `preprint`.

5. Frontmatter

There are two types of frontmatter coding:

(1) each author is connected to an affiliation with a footnote marker; hence all authors are grouped together and affiliations follow;

© 2019, Elsevier Ltd. Bugs, feature requests, suggestions and comments shall be mailed to <elsarticle@stmdocs.in>.

(2) authors of same affiliations are grouped together and the relevant affiliation follows this group.

An example of coding the first type is provided below.

```
\title{This is a specimen title\tnoteref{t1,t2}}
\tnotetext[t1]{This document is the results of the research
  project funded by the National Science Foundation.}
\tnotetext[t2]{The second title footnote which is a longer
  text matter to fill through the whole text width and
  overflow into another line in the footnotes area of the
  first page.}
```

```
\author[1]{Jos Migchielsen\corref{cor1}%
 \fnref{fn1}}
\ead{J.Migchielsen@elsevier.com}

\author[2]{CV Radhakrishnan\fnref{fn2}}
\ead{cvr@sayahna.org}

\author[3]{CV Rajagopal\fnref{fn1,fn3}}
\ead[url]{www.stmdocs.in}
```

```
\cortext[cor1]{Corresponding author}
\fntext[fn1]{This is the first author footnote.}
\fntext[fn2]{Another author footnote, this is a very long
  footnote and it should be a really long footnote. But this
  footnote is not yet sufficiently long enough to make two
  lines of footnote text.}
\fntext[fn3]{Yet another author footnote.}

\address[1]{Elsevier B.V., Radarweg 29, 1043 NX Amsterdam,
  The Netherlands}
\address[2]{Sayahna Foundations, JWRA 34, Jagathy,
  Trivandrum 695014, India}
\address[3]{STM Document Engineering Pvt Ltd., Mepukada,
  Malayinkil, Trivandrum 695571, India}
```

The output of the above TEX source is given in Clips 1 and 2. The header portion or title area is given in Clip 1 and the footer area is given in Clip 2.

QUICK LINKS

- Introduction
- Major Differences
- Installation
- Usage
- Frontmatter
- Floats
- Theorem and ...
- Enumerated ...
- Cross-references
- Mathematical ...
- Bibliography
- Graphical ...
- Final print

Clip 1: Header of the title page..

This is a specimen a_b title[★,★★]

Jos Migchielsen[a,1,*], CV Radhakrishnan[b,2], CV Rajagopal[c,1,3]

[a]*Elsevier B.V., Radarweg 29, 1043 NX Amsterdam, The Netherlands*
[b]*Sayahna Foundations, JWRA 34, Jagathy, Trivandrum 695014, India*
[c]*STM Document Engineering Pvt Ltd., Mepukada, Malayinkil, Trivandrum 695571, India*

Clip 2: Footer of the title page..

[illegible]

★This document is the results of the research project funded by the National Science Foundation.
★★The second title footnote which is a longer text matter to fill through the whole text width and overflow into another line in the footnotes area of the first page.
*Corresponding author
Email addresses: `J.Migchielsen@elsevier.com` (Jos Migchielsen), `cvr@sayahna.org` (CV Radhakrishnan)
URL: `www.stmdocs.in` (CV Rajagopal)
[1]This is the first author footnote.
[2]Another author footnote, this is a very long footnote and it should be a really long footnote. But this footnote is not yet sufficiently long enough to make two lines of footnote text.
[3]Yet another author footnote.

Most of the commands such as `\title`, `\author`, `\address` are self explanatory. Various components are linked to each other by a label–reference mechanism; for instance, title footnote is linked to the title with a footnote mark generated by referring to the `\label` string of the `\tnotetext`. We have used similar commands such as `\tnoteref` (to link title note to title); `\corref` (to link corresponding author text to corresponding author); `\fnref` (to link footnote text to the relevant author names). TeX needs two compilations to resolve the footnote marks in the preamble part. Given below are the syntax of various note marks and note texts.

```
\tnoteref{<label(s)>}
\corref{<label(s)>}
\fnref{<label(s)>}
\tnotetext[<label>]{<title note text>}
\cortext[<label>]{<corresponding author note text>}
\fntext[<label>]{<author footnote text>}
```

where `<label(s)>` can be either one or more comma delimited label strings. The optional arguments to the `\author` command holds the ref label(s) of the address(es) to which the author is affiliated while each `\address` command can have an optional argument of a label. In the same manner, `\tnotetext`, `\fntext`, `\cortext` will have optional arguments

as their respective labels and note text as their mandatory argument.

The following example code provides the markup of the second type of author-affiliation.

```
\author{Jos Migchielsen\corref{cor1}%
 \fnref{fn1}}
\ead{J.Migchielsen@elsevier.com}
 \address{Elsevier B.V., Radarweg 29, 1043 NX Amsterdam,
      The Netherlands}

\author{CV Radhakrishnan\fnref{fn2}}
\ead{cvr@sayahna.org}
 \address{Sayahna Foundations, JWRA 34, Jagathy,
   Trivandrum 695014, India}

\author{CV Rajagopal\fnref{fn1,fn3}}
\ead[url]{www.stmdocs.in}
  \address{STM Document Engineering Pvt Ltd., Mepukada,
   Malayinkil, Trivandrum 695571, India}
```

```
\cortext[cor1]{Corresponding author}
\fntext[fn1]{This is the first author footnote.}
\fntext[fn2]{Another author footnote, this is a very long
 footnote and it should be a really long footnote. But this
 footnote is not yet sufficiently long enough to make two lines
 of footnote text.}
```

The output of the above TEX source is given in Clip 3.

Clip 3: Header of the title page...

This is a specimen a_b title[⋆,⋆⋆]

Jos Migchielsen[1,*]

Elsevier B.V., Radarweg 29, 1043 NX Amsterdam, The Netherlands

CV Radhakrishnan[2]

Sayahna Foundations, JWRA 34, Jagathy, Trivandrum 695014, India

CV Rajagopal[1,3]

STM Document Engineering Pvt Ltd., Mepukada, Malayinkil, Trivandrum 695571, India

Clip 4 shows the output after giving `doubleblind` class option.

Clip 4: Double blind article.

This is a specimen a_b title

Abstract

In this work we demonstrate a_b the formation Y_1 of a new type of polariton

The frontmatter part has further environments such as abstracts and keywords. These can be marked up in the following manner:

```
\begin{abstract}
 In this work we demonstrate the formation of a new type of
 polariton on the interface between a ....
\end{abstract}
```

```
\begin{keyword}
 quadruple exiton \sep polariton \sep WGM
\end{keyword}
```

Each keyword shall be separated by a `\sep` command. MSC classifications shall be provided in the keyword environment with the commands `\MSC`. `\MSC` accepts an optional argument to accommodate future revisions. eg., `\MSC[2008]`. The default is 2000.

5.1. New page

Sometimes you may need to give a page-break and start a new page after title, author or abstract. Following commands can be used for this purpose.

```
 \newpageafter{title}
 \newpageafter{author}
 \newpageafter{abstract}
```

`\newpageafter{title}` typeset the title alone on one page.

`\newpageafter{author}` typeset the title and author details on one page.

`\newpageafter{abstract}` typeset the title, author details and abstract & keywords one one page.

6. Floats

Figures may be included using the command, `\includegraphics` in combination with or without its several options to further control graphic. `\includegraphics` is provided by graphic[s,x].sty which is part of any standard LaTeX distribution. graphicx.sty is loaded by default. LaTeX accepts figures in the postscript format while pdfLaTeX accepts *.pdf, *.mps (metapost), *.jpg and *.png formats. pdfLaTeX does not accept graphic files in the postscript format.

The `table` environment is handy for marking up tabular material. If users want to use multirow.sty, array.sty, etc., to fine control/enhance the tables, they are welcome to load any package of their choice and elsarticle.cls will work in combination with all loaded packages.

7. Theorem and theorem like environments

elsarticle.cls provides a few shortcuts to format theorems and theorem-like environments with ease. In all commands the options that are used with the `\newtheorem` command will work exactly in the same manner. elsarticle.cls provides three commands to format theorem or theorem-like environments:

```
\newtheorem{thm}{Theorem}
\newtheorem{lem}[thm]{Lemma}
\newdefinition{rmk}{Remark}
\newproof{pf}{Proof}
\newproof{pot}{Proof of Theorem \ref{thm2}}
```

The `\newtheorem` command formats a theorem in LaTeX's default style with italicized font, bold font for theorem heading and theorem number at the right hand side of the theorem heading. It also optionally accepts an argument which will be printed as an extra heading in parentheses.

```
\begin{thm}
 For system (8), consensus can be achieved with
 $\|T_{\omega z}$
 ...
   \begin{eqnarray}\label{10}
   ....
   \end{eqnarray}
\end{thm}
```

Clip 5 will show you how some text enclosed between the above code

© 2019, Elsevier Ltd. Bugs, feature requests, suggestions and comments shall be mailed to <elsarticle@stmdocs.in>.

looks like:

Clip 5: `\newtheorem`.

Theorem 2. *For system (8), consensus can be achieved with* $\|T_{\omega z}(s)\|_\infty < \gamma$ *if there exist a symmetric positive definite matrix* $P \in \mathcal{R}^{(n-1)\times(n-1)}$ *and a scalar* $\mu > 0$ *satisfying*

$$\Gamma = \begin{bmatrix} -\bar{L}^T P - P\bar{L} + U_1^T U_1 + \mu\bar{E} & PU_1^T E_1 & PU_1^T \\ E_1^T U_1 P & -\mu I & 0 \\ U_1 P & 0 & -\gamma^2 I \end{bmatrix} < 0, \tag{10}$$

where $\bar{L} = U_1^T L U_1$ *and* $\bar{E} = U_1^T E_2^T E_2 U_1$.

The `\newdefinition` command is the same in all respects as its `\newtheorem` counterpart except that the font shape is roman instead of italic. Both `\newdefinition` and `\newtheorem` commands automatically define counters for the environments defined.

Clip 6: `\newdefinition`.

Remark 3. We remark that; when the ratio h/λ tends to 0, the expression $\lambda L(r,s) = -(s-r)/(4\left(\frac{h}{\lambda}\right)^2 + (r-s)^2)$ tends to $1/(r-s)$ which is a singular function. This means that the expression $\lambda L(r,s)$ is not well behaved for the small values of h/λ. Consequently, for the solution to converge, the integrals of (10) and (11) must be evaluated with a large number of nodes. In our numerical applications (cf. section 5), we use 100 nodes to evaluate these integrals. With the smallest value of $h/\lambda = 0.02$, the convergence is good with $N = 20$.

The `\newproof` command defines proof environments with upright font shape. No counters are defined.

Clip 7: `\newproof`.

Proof of Theorem 2. Proof follows straightforward from Lemma 3 and Theorem 1. However, it should be emphasized that all possible $\bar{L}_{\sigma(t)}$ should share a common Lyapunov function $V(\delta) = \delta^T(t)P\delta(t)$ (see the proof of Lemma 3 in Appendix A). □

Users can also make use of `amsthm.sty` which will override all the default definitions described above.

8. Enumerated and Itemized Lists

elsarticle.cls provides an extended list processing macros which makes the usage a bit more user friendly than the default LaTeX list macros. With an optional argument to the `\begin{enumerate}` command, you can change the list counter type and its attributes.

```
\begin{enumerate}[1.]
\item The enumerate environment starts with an optional
  argument ‘1.’, so that the item counter will be suffixed
  by a period.
\item You can use ‘a)’ for alphabetical counter and ’(i)’ for
  roman counter.
 \begin{enumerate}[a)]
  \item Another level of list with alphabetical counter.
  \item One more item before we start another.
```

Clip 8: List – Enumerate.

1. The enumerate environment starts with an optional argument ‘1.’ so that the item counter will be suffixed by a period.
2. You can use ‘(a)’ for alphabetical counter and ’(i)’ for roman counter.
    a) Another level of list with alphabetical counter.
    b) One more item before we start another.
        (i) This item has roman numeral counter.
        (ii) Another one before we close the third level.
    c) Third item in second level.
3. All list items conclude with this step.

Further, the enhanced list environment allows one to prefix a string like ‘step’ to all the item numbers.

```
\begin{enumerate}[Step 1.]
 \item This is the first step of the example list.
 \item Obviously this is the second step.
 \item The final step to wind up this example.
\end{enumerate}
```

Clip 9: List – enhanced.

Step 1. This is the first step of the example list.
Step 2. Obviously this is the second step.
Step 3. The final step to wind up this example.

9. Cross-references

In electronic publications, articles may be internally hyperlinked. Hyperlinks are generated from proper cross-references in the article. For example, the words Fig. 1 will never be more than simple text, whereas the proper cross-reference `\ref{tiger}` may be turned into a hyperlink to the figure itself: Fig. 1. In the same way, the words Ref. [1] will fail to turn into a hyperlink; the proper cross-reference is `\cite{Knuth96}`. Cross-referencing is possible in LaTeX for sections, subsections, formulae, figures, tables, and literature references.

10. Mathematical symbols and formulae

Many physical/mathematical sciences authors require more mathematical symbols than the few that are provided in standard LaTeX. A useful package for additional symbols is the amssymb package, developed by the American Mathematical Society. This package includes such oft-used symbols as $\lesssim$ (`\lesssim`), $\gtrsim$ (`\gtrsim`) or $\hbar$ (`\hbar`). Note that your TeX system should have the msam and msbm fonts installed. If you need only a few symbols, such as $\Box$ (`\Box`), you might try the package latexsym.

Another point which would require authors' attention is the breaking up of long equations. When you use elsarticle.cls for formatting your submissions in the `preprint` mode, the document is formatted in single column style with a text width of 384pt or 5.3in. When this document is formatted for final print and if the journal happens to be a double column journal, the text width will be reduced to 224pt at for 3+ double column and 5+ journals respectively. All the nifty fine-tuning in equation breaking done by the author goes to waste in such cases. Therefore, authors are requested to check this problem by typesetting their submissions in final format as well just to see if their equations are broken at appropriate places, by changing appropriate options in the document class loading command, which is explained in section 4, Usage. This allows authors to fix any equation breaking problem before submission for publication. elsarticle.cls supports formatting the author submission in different types of final format. This is further discussed in section 13, Final print.

Displayed equations and double column journals

Many Elsevier journals print their text in two columns. Since the preprint layout uses a larger line width than such columns, the formulae are too wide for the line width in print. Here is an example of an equation (see equation 6) which is perfect in a single column preprint format:

© 2019, Elsevier Ltd. Bugs, feature requests, suggestions and comments shall be mailed to <elsarticle@stmdocs.in>.

Clip 10: See equation (6).

Here e, m are the electron charge and mass; $\mathbf{p}$ is the electron momentum. For the quadrupole $1S$ transition in cuprous oxide the energy of interaction can be written as:

$$\sum_{i=0}^{\infty} An \int \mathrm{d}x \frac{F_n(x)}{A_n + B_n} = B^n C^n \int \mathrm{d}x \int \mathrm{d}y \frac{G_n(x,y)}{A_n x + \mathcal{B}_n y} + \frac{G_n(x,y)}{A_n x + \mathcal{B}_n y} \tag{5}$$

Here we introduced the initial state of the system, which transforms as irreducible representation $^1\Gamma_1^+$ of the cubic centered group O_h. The final state is the *ortho*-exciton state which transforms as $^3\Gamma_{5,xz}^+$ in Cartesian system or as $^3\Gamma_{5;1,2}^+$ in the corresponding spherical basis.

Hence, using (1, 3, 4, 5), one can deduce that the the coupling of the spherical harmonic compared to the plane wave ($\hbar g_{1,2} = 124\ \mu eV$) is resonantly enhanced:

$$\frac{g_{1,39}}{g_{1,2}} = -i0.06 b_{1,39} (kr_0) A_{1,39}^{1,2} (r_0 + \delta r) \tag{6}$$

Here we utilized the fact that $B_{1,39}^{1,2} \ll A_{1,39}^{1,2}$. While the resonant enhancement is provided by the $b_{1,39}$ Mie coefficient here, the translational coefficient reduces the effect. That is why if one tries to couple the evanescent light to the dipole transition the effect is much weaker as $A_{1,39}^{0,1} \ll A_{1,39}^{1,2}$.

When this document is typeset for publication in a model 3+ journal with double columns, the equation will overlap the second column text matter if the equation is not broken at the appropriate location.

Clip 11: See equation (6) overprints into second column.

Their explicit expression can be found, for instance, in [12, 9] and are explicitly listed in the Appendix.

The bulk (incident) and evanescent polaritons in cuprous oxide are formed through the quadrupole part of the light-matter interaction:

$$H_{int} = \frac{ie}{m\omega_{1S}} \mathbf{E}_{i,s} \cdot \mathbf{p}$$

Here e, m are the electron charge and mass; $\mathbf{p}$ is the electron momentum. For the quadrupole $1S$ transition in cuprous oxide the energy of interaction can be written as:

$$\sum_{i=0}^{\infty} An \int \mathrm{d}x \frac{F_n(x)}{A_n + B_n} = B^n C^n \int \mathrm{d}x \int \mathrm{d}y \frac{G_n(x,y)}{A_n x + \mathcal{B}_n y} + \frac{G_n(x,y)}{A_n x + \mathcal{B}_n y} \tag{5}$$

Here we introduced the initial state of the system, which transforms as irreducible representation $^1\Gamma_1^+$ of the cubic centered group O_h. The final state is the *ortho*-exciton

Figure 1: The evanescent light - $1S$ quadrupole coupling ($g_{1,l}$) scaled to the bulk exciton-photon coupling ($g_{1,2}$). The size parameter kr_0 is denoted as x and the PMS is placed directly on the cuprous oxide sample ($\delta r = 0$, See also Fig.2).

3. Results and discussion

In this section let us utilize the above calculated WGM-QE interaction to obtain the evanescent polariton (EP) dispersion in the framework of the coupled oscillator model that has been widely used for describing coupled atom-photon or exciton-photon modes in microcavity systems [13]. Near the resonance between WGM and the quadrupole exciton $\omega_{1l} \approx \omega_{1S}$ the EP branches are given by the eigenvalues of the following Hamiltonian:

$$H/\hbar = \omega_{1l} a_x^\dagger a_x + \omega_{1S} b_x^\dagger b_x + g_{1l}(x) \left(a_k^\dagger b_x + a_x b_x^\dagger \right), \tag{7}$$

The typesetter will try to break the equation which need not necessarily be to the liking of the author or as it happens, typesetter's break point may be semantically incorrect. Therefore, authors may check their submissions for the incidence of such long equations and break the equations at the correct places so that the final typeset copy will be as they wish.

11. Bibliography

Three bibliographic style files (`*.bst`) are provided — elsarticle-num.bst, elsarticle-num-names.bst and elsarticle-harv.bst — the first one can be used for the numbered scheme, second one for numbered with new options of natbib.sty. The third one is for the author year scheme.

In LaTeX literature, references are listed in the `thebibliography` environment. Each reference is a `\bibitem` and each `\bibitem` is identified by a label, by which it can be cited in the text:

`\bibitem[Elson et al.(1996)]{ESG96}` is cited as `\citet{ESG96}`.

In connection with cross-referencing and possible future hyperlinking it is not a good idea to collect more that one literature item in one `\bibitem`. The so-called Harvard or author-year style of referencing is enabled by the LaTeX package natbib. With this package the literature can be cited as follows:

- Parenthetical: `\citep{WB96}` produces (Wettig & Brown, 1996).
- Textual: `\citet{ESG96}` produces Elson et al. (1996).
- An affix and part of a reference: `\citep[e.g.][Ch. 2]{Gea97}` produces (e.g. Governato et al., 1997, Ch. 2).

In the numbered scheme of citation, `\cite{<label>}` is used, since `\citep` or `\citet` has no relevance in the numbered scheme. natbib package is loaded by elsarticle with `numbers` as default option. You can change this to author-year or harvard scheme by adding option `authoryear` in the class loading command. If you want to use more options of the natbib package, you can do so with the `\biboptions` command, which is described in the section 4, Usage. For details of various options of the natbib package, please take a look at the natbib documentation, which is part of any standard LaTeX installation.

In addition to the above standard `.bst` files, there are 10 journal-specific `.bst` files also available. Instruction for using these `.bst` files can be found at http://support.stmdocs.in

12. Graphical abstract and highlights

A template for adding graphical abstract and highlights are available now. This will appear as the first two pages of the PDF before the article content begins.

© 2019, Elsevier Ltd. Bugs, feature requests, suggestions and comments shall be mailed to <elsarticle@stmdocs.in>.

Please refer below to see how to code them.

```
....
....

\end{abstract}

%%Graphical abstract
\begin{graphicalabstract}
%\includegraphics{grabs}
\end{graphicalabstract}

%%Research highlights
\begin{highlights}
\item Research highlight 1
\item Research highlight 2
\end{highlights}

\begin{keyword}
%% keywords here, in the form: keyword \sep keyword
....
....
```

13. Final print

The authors can format their submission to the page size and margins of their preferred journal. elsarticle provides four class options for the same. But it does not mean that using these options you can emulate the exact page layout of the final print copy.

`1p`: 1+ journals with a text area of 384pt × 562pt or 13.5cm × 19.75cm or 5.3in × 7.78in, single column style only.

`3p`: 3+ journals with a text area of 468pt × 622pt or 16.45cm × 21.9cm or 6.5in × 8.6in, single column style.

`twocolumn`: should be used along with 3p option if the journal is 3+ with the same text area as above, but double column style.

`5p`: 5+ with text area of 522pt × 682pt or 18.35cm × 24cm or 7.22in × 9.45in, double column style only.

Following pages have the clippings of different parts of the title page of different journal models typeset in final format.

Model 1+ and 3+ will have the same look and feel in the typeset copy when presented in this document. That is also the case with the double column 3+ and 5+ journal article pages. The only difference will be wider text width of higher models. Therefore we will look at the different portions of a typical single column journal page and that of a double column article in the final format.

[Specimen single column article – Click here]
[Specimen double column article – Click here]

© 2019, Elsevier Ltd. Bugs, feature requests, suggestions and comments shall be mailed to <elsarticle@stmdocs.in>.

QUICK LINKS

▶ Introduction ▶ Major Differences
▶ Installation ▶ Usage
▶ Frontmatter ▶ Floats
▶ Theorem and ... ▶ Enumerated ...
▶ Cross-references ▶ Mathematical ...
▶ Bibliography ▶ Graphical ...
▶ Final print

This is a specimen a_b title[⋆,⋆⋆]

Jos Migchielsen[1,∗]

Elsevier B.V., Radarweg 29, 1043 NX Amsterdam, The Netherlands

CV Radhakrishnan[2]

Sayahna Foundations, JWRA 34, Jagathy, Trivandrum 695014, India

CV Rajagopal[1,3]

STM Document Engineering Pvt Ltd., Mepukada, Malayinkil, Trivandrum 695571, India

Abstract

In this work we demonstrate a_b the formation Y_1 of a new type of polariton on the interface between a cuprous oxide slab and a polystyrene micro-sphere placed on the slab. The evanescent field of the resonant whispering gallery mode (WGM) of the micro sphere has a substantial gradient, and therefore effectively couples with the quadrupole $1S$ excitons in cuprous oxide. This evanescent polariton has a long life-time, which is determined only by its excitonic and WGM component. The polariton lower branch has a well pronounced minimum. This suggests that this excitation is localized and can be utilized for possible BEC. The spatial coherence of the polariton can be improved by assembling the micro-spheres into a linear chain.

Keywords: quadrupole exciton, polariton, WGM, BEC
JEL: 71.35.-y, 71.35.Lk, 71.36.+c

1. Introduction

Although quadrupole excitons (QE) in cuprous oxide crystals are good candidates for BEC due to their narrow line-width and long life-time there are some factors impeding BEC [1, 2]. One of these factors is that due to the small but non negligible coupling to the photon bath, one must consider BEC of the corresponding mixed light-matter states called polaritons [3]. The photon-like part of the polariton has a large group velocity and tends to escape from the crystal. Thus,

⋆This document is the results of the research project funded by the National Science Foundation.

⋆⋆The second title footnote which is a longer text matter to fill through the whole text width and overflow into another line in the footnotes area of the first page.

∗Corresponding author
Email addresses: `J.Migchielsen@elsevier.com` (Jos Migchielsen), `cvr@sayahna.org` (CV Radhakrishnan)
URL: `www.stmdocs.in` (CV Rajagopal)

[1]This is the first author footnote.

[2]Another author footnote, this is a very long footnote and it should be a really long footnote. But this footnote is not yet sufficiently long enough to make two lines of footnote text.

[3]Yet another author footnote.

Preprint submitted to Elsevier *June 8, 2018*

This is a specimen a_b title[⋆,⋆⋆]

Jos Migchielsen[1,*]

Elsevier B.V., Radarweg 29, 1043 NX Amsterdam, The Netherlands

CV Radhakrishnan[2]

Sayahna Foundations, JWRA 34, Jagathy, Trivandrum 695014, India

CV Rajagopal[1,3]

STM Document Engineering Pvt Ltd., Mepukada, Malayinkil, Trivandrum 695571, India

Abstract

In this work we demonstrate a_b the formation Y_1 of a new type of polariton on the interface between a cuprous oxide slab and a polystyrene micro-sphere placed on the slab. The evanescent field of the resonant whispering gallery mode (WGM) of the micro sphere has a substantial gradient, and therefore effectively couples with the quadrupole $1S$ excitons in cuprous oxide. This evanescent polariton has a long life-time, which is determined only by its excitonic and WGM component. The polariton lower branch has a well pronounced minimum. This suggests that this excitation is localized and can be utilized for possible BEC. The spatial coherence of the polariton can be improved by assembling the micro-spheres into a linear chain.

Keywords: quadrupole exciton, polariton, WGM, BEC
JEL: 71.35.-y, 71.35.Lk, 71.36.+c

1. Introduction

Although quadrupole excitons (QE) in cuprous oxide crystals are good candidates for BEC due to their narrow line-width and long life-time there are some factors impeding BEC [1, 2]. One of these factors is that due to the small but non negligible coupling to the photon bath, one must consider BEC of the corresponding mixed light-matter states called polaritons [3]. The photon-like part of the polariton has a large group velocity and tends to escape from the crystal. Thus, the temporal coherence of the condensate is effectively broken [4, 5]. One proposed solution to this issue is to place the crystal into a planar micro-cavity [6]. But even state-of-the-art planar micro-cavities can hold the light no longer than 10 μs. Besides, formation of the polaritons in the planar cuprous oxide micro-cavity is not effective due to quadrupole origin of the excitons.

Theorem 1. *In this work we demonstrate the formation of a new type of polariton on the interface between a cuprous oxide slab and a polystyrene micro-sphere placed on the slab. The evanescent field of the resonant whispering gallery mode (WGM) of the micro sphere has a substantial gradient, and therefore effectively couples with the quadrupole $1S$ excitons in cuprous oxide. This evanescent polariton has a long life-time, which is determined only by its excitonic and WGM component. The polariton lower branch has a well pronounced minimum. This suggests that this excitation is localized and can be utilized for possible BEC. The spatial coherence of the polariton can be improved by assembling the micro-spheres into a linear chain.*

Therefore in this work we propose to prevent the polariton escaping by trapping it into a whispering gallery mode (WGM)[4] of a polystyrene micro-sphere (PMS).

We develop a model which demonstrates formation of a strongly *localized* polariton-like quasi-particle. This quasi-particle is formed by the *resonant* interaction between the WGM in PMS and QE in the adjacent layer of cuprous oxide. The QE interacts with the *gradient* of the WGM evanescent field.

⋆This document is the results of the research project funded by the National Science Foundation.
⋆⋆The second title footnote which is a longer text matter to fill through the whole text width and overflow into another line in the footnotes area of the first page.

*Corresponding author
Email addresses: J.Migchielsen@elsevier.com (Jos Migchielsen), cvr@sayahna.org (CV Radhakrishnan)
URL: www.stmdocs.in (CV Rajagopal)
[1]This is the first author footnote.
[2]Another author footnote, this is a very long footnote and it should be a really long footnote. But this footnote is not yet sufficiently long enough to make two lines of footnote text.
[3]Yet another author footnote.

[4]WGM occur at particular resonant wavelengths of light for a given dielectric sphere size. At these wavelengths, the light undergoes total internal reflection at the sphere surface and becomes trapped within the particle for timescales of the order of ns.

© 2019, Elsevier Ltd. Bugs, feature requests, suggestions and comments shall be mailed to <elsarticle@stmdocs.in>.
