# Supplementary material for: Caretta – A multiple protein structure alignment and feature extraction suite
Source: Comput Struct Biotechnol J. 2020 Apr 6;18:981–92. doi: 10.1016/j.csbj.2020.03.011 (PMC7186369; doi:10.1016/j.csbj.2020.03.011)
Supplement: Supplementary file 1 [file mmc1.zip › doc/elstest-3pd.pdf]

This is a specimen a_b title[⋆,⋆⋆]

Jos Migchielsen[a,1,*], CV Radhakrishnan[b,2], CV Rajagopal[c,1,3]

[a] *Elsevier B.V., Radarweg 29, 1043 NX Amsterdam, The Netherlands*
[b] *Sayahna Foundations, JWRA 34, Jagathy, Trivandrum 695014, India*
[c] *STM Document Engineering Pvt Ltd., Mepukada, Malayinkil, Trivandrum 695571, India*

Abstract

In this work we demonstrate a_b the formation Y_1 of a new type of polariton on the interface between a cuprous oxide slab and a polystyrene micro-sphere placed on the slab. The evanescent field of the resonant whispering gallery mode (WGM) of the micro sphere has a substantial gradient, and therefore effectively couples with the quadrupole $1S$ excitons in cuprous oxide. This evanescent polariton has a long life-time, which is determined only by its excitonic and WGM component. The polariton lower branch has a well pronounced minimum. This suggests that this excitation is localized and can be utilized for possible BEC. The spatial coherence of the polariton can be improved by assembling the micro-spheres into a linear chain.

Keywords: quadrupole exciton, polariton, WGM, BEC
JEL: 71.35.-y, 71.35.Lk, 71.36.+c

1. Introduction

Although quadrupole excitons (QE) in cuprous oxide crystals are good candidates for BEC due to their narrow line-width and long life-time there are some factors impeding BEC [1, 2]. One of these factors is that due to the small but non negligible coupling to the photon bath, one must consider BEC of the corresponding mixed light-matter states called polaritons [3]. The photon-like part of the polariton has a large group velocity and tends to escape from the crystal. Thus, the temporal coherence of the condensate is effectively broken [4, 5]. One proposed solution to this issue is to place the crystal into a planar micro-cavity [6]. But even state-of-the-art planar micro-cavities can hold the light no longer than 10 μs. Besides, formation of the polaritons in the planar cuprous oxide micro-cavity is not effective due to quadrupole origin of the excitons.

Theorem 1. *In this work we demonstrate the formation of a new type of polariton on the interface between a cuprous oxide slab and a polystyrene micro-sphere placed on the slab. The evanescent field of the resonant whispering gallery mode (WGM) of the micro sphere has a substantial gradient, and therefore effectively couples with the quadrupole $1S$ excitons in cuprous oxide. This evanescent polariton has a long life-time, which is determined only by its excitonic and WGM component. The polariton lower branch has a well pronounced minimum. This suggests that this excitation is localized and can be utilized for possible BEC. The spatial coherence of the polariton can be improved by assembling the micro-spheres into a linear chain.*

Therefore in this work we propose to prevent the polariton escaping by trapping it into a whispering gallery mode (WGM)[4] of a polystyrene micro-sphere (PMS).

We develop a model which demonstrates formation of a strongly *localized* polariton-like quasi-particle.

[⋆] This document is the results of the research project funded by the National Science Foundation.

[⋆⋆] The second title footnote which is a longer text matter to fill through the whole text width and overflow into another line in the footnotes area of the first page.

[*] Corresponding author

Email addresses: `J.Migchielsen@elsevier.com` (Jos Migchielsen), `cvr@sayahna.org` (CV Radhakrishnan)

URL: `www.stmdocs.in` (CV Rajagopal)

[1] This is the first author footnote.

[2] Another author footnote, this is a very long footnote and it should be a really long footnote. But this footnote is not yet sufficiently long enough to make two lines of footnote text.

[3] Yet another author footnote.

[4] WGM occur at particular resonant wavelengths of light for a given dielectric sphere size. At these wavelengths, the light undergoes total internal reflection at the sphere surface and becomes trapped within the particle for timescales of the order of ns.

This quasi-particle is formed by the *resonant* interaction between the WGM in PMS and QE in the adjacent layer of cuprous oxide. The QE interacts with the *gradient* of the WGM evanescent field.

There are few experiments concerned with resonant interaction of the WGM and dipole allowed exciton (DE) [7, 8]. But the DE has some disadvantages compared to QE when it comes to interaction with the WGM. First, the evanescent light has small intensity. Therefore it is not effective for the dipole allowed coupling. But it has a large gradient, so it can effectively couple through a quadrupole part. Second, the DE has short life time compared to the QE and therefore is not suitable for BEC. Third, the kinetic energy of the DE is comparable with the interaction energy. Hence the localization is effectively impeded.

2. Evanescent vs. conventional quadrupole light-matter coupling

Assume that a *single* PMS of radius r_0 μm is placed at a small [5] distance $\delta r_0 \ll r_0$ from the cuprous oxide crystal ($\epsilon_{Cu2O} = 6.5$).

There are several methods to observe WGM-QE interaction. One of them is to mount a prism (or a fiber) on the top of PMS [7]. But any surface nearby perturbs spherical symmetry and therefore reduces the Q factor and the life-time of the WGM. But the positions of the corresponding Mie resonances are not affected. Therefore in this paper we adopt a slightly different picture. Namely the scattering of the bulk polariton in cuprous oxide by the PMS. If one of the Mie resonances is in resonance with the QE one can expect formation of the new type of polariton.

Assume some density of quadrupole $1S$ excitons ([QE]), $\hbar\omega_{1S} = 2.05\ eV$, $\lambda_{1S} = 2\pi/\omega_{1S} = 6096$ Å) has been created by an external laser pulse. The corresponding polaritons move in the crystal as the polariton and can be trapped by the PMS due to WGM-QE resonant interaction.

The WGM evanescent field penetration depth into the cuprous oxide adjacent crystal is much larger than the QE radius:

$$\lambda_{1S}/2\pi\,(\epsilon_{Cu2O} - 1)^{1/2} = 414\ \text{Å} \gg a_B = 4.6\ \text{Å}$$

Hence, the light-matter interaction can be considered semi-classically. For the same reason we consider only *bulk* polaritons. However the theory may be expanded to include the surface polaritons also. In the late case the evanescent field of such a surface polariton could play an essential role in the interaction with the WGM, comparable with WGM-QE coupling.

For resonance coupling with a WGM its size parameter should be determined by the resonant wave vector in the cuprous oxide $k_0 = 2.62 \times 10^7\ m^{-1}$. For example, if one takes a polystyrene (refractive index $\epsilon^2 = 1.59$) sphere of radius $r_0 = 10.7\ \mu m$ then $k_0 r_0 = 28.78350$. This size parameter corresponds to the 39TE1 resonance [9].

The photon part of the polariton trapped inside the PMS moves as it would move in a micro-cavity of the effective modal volume $V \ll 4\pi r_0^3/3$. Consequently, it can escape through the evanescent field. This evanescent field essentially has a quantum origin and is due to tunneling through the potential caused by dielectric mismatch on the PMS surface. Therefore, we define the *evanescent* polariton (EP) as an evanescent light - QE coherent superposition.

Below we compare the evanescent quadrupole polariton and conventional bulk quadrupole polariton in cuprous oxide. For simplicity let us consider the incident polariton wave vector running along the interface (z direction). The polarization of the polariton is taken along the x direction. Therefore, in the system of coordinates centered at the sphere, the photon part of the incident polariton can be written as [10]:

$$\mathbf{E}_i = \sum_l E_0 i^l \frac{2l+1}{l\,(l+1)} \left(\mathbf{M}_{1l} - i\mathbf{N}_{1l}\right), \tag{1}$$

where $\mathbf{M}_{1l}$ and $\mathbf{N}_{1l}$ are vector spherical harmonics corresponding to TE- and TM- polarized modes of angular momentum l; the z component of the angular momentum is $|m| = 1$; E_0 is the amplitude of the electric field. The scattered field is given as:

$$\mathbf{E}_s = \sum_l E_0 i^l \frac{2l+1}{l\,(l+1)} \left(i a_{1l}\mathbf{N}_{1l} - b_{1l}\mathbf{M}_{1l}\right), \tag{2}$$

here a_{1l} and b_{1l} are scattering Mie coefficients (See the Appendix). Taking into account that both WGM and QE have narrow line-width, and the energy separation between different WGM is much bigger then the interaction energy we adopt a single mode picture [7]. Keeping only the resonant term the last expression yields:

$$\mathbf{E}_s = -E_0 i^l 0.05 b_{1,39}\mathbf{M}_{1,39}, \tag{3}$$

To calculate the interaction of the plane wave (conventional polariton) (1) and WGM (evanescent polariton) with cuprous oxide one has to change to the cuprous

[5] comparing to the evanescent field penetration depth

oxide centered system of coordinate (See Fig.2) While in the system of the coordinate, centered at the cuprous oxide, the plane wave is still given by the expression (1), the scattered field has to be changed according to the vector spherical harmonic addition theorem [11]:

$$\mathbf{M}_{1,39} = A^{ml}_{1,39}\left(r_0 + \delta r\right)\mathbf{M}_{ml} + B^{ml}_{1,39}\left(r_0 + \delta r\right)\mathbf{N}_{ml} \quad (4)$$

Here $A^{ml}_{1,39}$ and $B^{ml}_{1,39}$ are the translational coefficients. Their explicit expression can be found, for instance, in [12, 9] and are explicitly listed in the Appendix.

The bulk (incident) and evanescent polaritons in cuprous oxide are formed through the quadrupole part of the light-matter interaction:

$$H_{int} = \frac{ie}{m\omega_{1S}}\mathbf{E}_{i,s}\cdot\mathbf{p}$$

Here e, m are the electron charge and mass; $\mathbf{p}$ is the electron momentum. For the quadrupole $1S$ transition in cuprous oxide the energy of interaction can be written as:

$$\sum_{i=0}^{\infty} An \int \mathrm{d}x \frac{F_n(x)}{A_n + B_n} = B^n C^n \int \mathrm{d}x \int \mathrm{d}y \frac{G_n(x,y)}{A_n x + \mathcal{B}_n y} + \frac{G_n(x,y)}{A_n x + \mathcal{B}_n y} \quad (5)$$

Here we introduced the initial state of the system, which transforms as irreducible representation ${}^1\Gamma_1^+$ of the cubic centered group O_h. The final state is the *ortho*-exciton state which transforms as ${}^3\Gamma^+_{5,xz}$ in Cartesian system or as ${}^3\Gamma^+_{5;1,2}$ in the corresponding spherical basis.

Hence, using (1, 3, 4, 5), one can deduce that the the coupling of the spherical harmonic compared to the plane wave ($\hbar g_{1,2} = 124\,\mu eV$) is resonantly enhanced:

$$\frac{g_{1,39}}{g_{1,2}} = -i0.06 b_{1,39}\left(kr_0\right) A^{1,2}_{1,39}\left(r_0 + \delta r\right) \quad (6)$$

Here we utilized the fact that $B^{1,2}_{1,39} \ll A^{1,2}_{1,39}$. While the resonant enhancement is provided by the $b_{1,39}$ Mie coefficient here, the translational coefficient reduces the effect. That is why if one tries to couple the evanescent light to the dipole transition the effect is much weaker as $A^{0,1}_{1,39} \ll A^{1,2}_{1,39}$. The resulting exciton - evanescent light coupling is shown in Fig.1 Both dipole and quadrupole coupling rate in the actual combined semiconductor-microsphere system is smaller then that in case of conventional polariton. This is attributed to the fact that the coupling occurs in a small region of the evanescent tail penetrating into cuprous oxide, although the coupling grows with mode number l, because the gradient of the evanescent field increases. Note that QE realizes *strong* coupling regime $g_{1,39} > \gamma$ while DE demonstrates *weak* regime only [7]. The property of the scalable coupling factor can be utilized in practical applications such as non-linear optics and is the subject of our future work.

Figure 1: The evanescent light - $1S$ quadrupole coupling ($g_{1,l}$) scaled to the bulk exciton-photon coupling ($g_{1,2}$). The size parameter kr_0 is denoted as x and the PMS is placed directly on the cuprous oxide sample ($\delta r = 0$, See also Fig.2).

3. Results and discussion

In this section let us utilize the above calculated WGM-QE interaction to obtain the evanescent polariton (EP) dispersion in the framework of the coupled oscillator model that has been widely used for describing coupled atom-photon or exciton-photon modes in microcavity systems [13]. Near the resonance between WGM and the quadrupole exciton $\omega_{1l} \approx \omega_{1S}$ the EP branches are given by the eigenvalues of the following Hamiltonian:

$$H/\hbar = \omega_{1l} a^\dagger_x a_x + \omega_{1S} b^\dagger_x b_x + g_{1l}\left(x\right)\left(a^\dagger_k b_x + a_x b^\dagger_x\right), \quad (7)$$

here a_x, b_x are annihilation operators for light and the exciton, respectively. We also neglected kinetic energy of the QE due to smallness of the resonant wave vector and big mass of the QE. Therefore, considering that both the exciton and WGM of a single sphere are localized, the dispersion is reduced to:

$$\omega = \omega_{1S} \pm g_{1l}/\hbar \quad (8)$$

The above expression shows the formation of the doublet at resonance (both states are exactly half-QE, half-WGM). Recall that for DE-WGM weak coupling only WGM pattern shifted by the coupling has been observed [7].

The excitons are trapped in the minimum of the lower branch thus populating the strongly localized states. Physically this means that the resonant coupling with localized WGM does not let QE escape by means of its kinetic energy.

The dispersion above is similar to the quadrupole-dipole hybrid in the organic-inorganic hetero-structures [2]. In the later case, the excited organic molecules create an evanescent field penetrating into the cuprous oxide.

Now let us consider possible application of the evanescent polariton to BEC. The problem of the conventional polariton escaping from the crystal mentioned

in the introduction no longer exists for the localized states of the evanescent polariton.

The linewidth of the mixed state is expected to be in the first approximation the sum of the exciton and photon linewidth weighted by the exciton and photon component of the polariton. At resonance, the linewidth of the two eigenstates is simply given by $\gamma = (\gamma_{1S} + \gamma_{m,l})/2$. Where γ_{1S} and $\gamma_{m,l}$ are the QE and WGM linewidth. Even having taken into account decrease of the Q factor due to PMS contact with the cuprous oxide sample $\gamma_{1S} \gg \gamma_{1,39}$ for the ortho-exciton. Hence, γ is defined by the QE linewidth.

For the Cu_2O para-exciton the linewidth of the polariton is given by the WGM linewidth. The para-excitons can acquire some oscillator strength provided the PMS exerts a local stress upon the cuprous oxide sample. The applied stress changes the crystal symmetry, so that usually optically inactive para-excitons may couple to the WGM. Note that due to spin-orbit interaction the para-excitons are 12 μeV below the ortho-exciton. Therefore for given radius of the PMS the resonant interaction occurs with different WGM for ortho- and para- cases.

The evanescent polariton provided by a single sphere gives the time coherence necessary for the observable BEC of the quadrupole exciton. But the spatial coherence is limited to a small region near the sphere. To improve the spatial coherence one has to sacrifice the temporal coherence slightly by delocalizing the corresponding WGM. It can be done by using an array of spheres aligned along the z direction and separated by the distance δr_0 (See Fig.2).

Figure 2: Schematic of formation of the evanescent polariton on linear chain of PMS. The actual dispersion is determined by the ratio of two coupling parameters such as exciton-WGM coupling and WGM-WGM coupling between the microspheres.

Recent experimental [14] and theoretical [15] studies have shown that the WGM can travel along the chain as "heavy photons". Therefore the WGM acquires the spatial dispersion, and the evanescent quadrupole polariton has the form (See Fig.3):

$$2\omega = \omega_{1l,k} + \omega_{1S} \pm \sqrt{(\omega_{1l,k} - \omega_{1S})^2 + 4|g_{1l}/\hbar|^2}$$
$$\omega_{1l,k} = \omega_{1S} + 2\left(g_{1l}^{1l}/\hbar\right) cos(x - x_{1l} + \pi/2) \quad (9)$$

Here $g_{1l}^{1l} = \omega_{1S} b_{1l} A_{1l}^{1l}(\delta r_1)$ is the nearest-neighbor inter-sphere coupling parameter.

Figure 3: Dispersion of the evanescent polariton 39TE1. The dashed line (1) corresponds to the dispersion of the chain of spheres touching each other ($\delta r_0 = 0$). The thin solid line (3) stands for upper and lower branches of a single sphere dispersion ($\delta r_0 \gg \delta r = 0$). The thick solid curve (2) is the case of linear chain of the spheres in contact with the cuprous oxide ($\delta r_0 = \delta r = 0$).

When the coupling between spheres dominates ($\delta r \gg \delta r_0$) the minimum of the lower polariton branch disappears. Consequently, for possible BEC of the evanescent polariton one has to keep the desired balance between spatial and temporal coherence by adjusting experimental parameters δr and δr_0.

Both, the energy of the $1S$ quadrupole exciton and the WGM depend on the temperature. Therefore one can use a standard temperature scan to reveal the evanescent polariton dispersion [16].

In summary, we note that there is some similarity between BEC of alkali atoms trapped by the laser field [17] and the long living QE localized by the resonant WGM.

The theory developed above is applicable also for void cavities, spherical impurities and metallic droplets in bulk cuprous oxide crystal.

4. Appendix

In the appendix we list explicit expression for the Mie scattering coefficient:

$$a_{ml} = \frac{n^2 j_{ml}(nx)\left[x j_{ml}(x)\right]' - j_{ml}(x)\left[nx j_{ml}(nx)\right]'}{n^2 j_{ml}(nx)\left[x h_{ml}^{(1)}(x)\right]' - h_{ml}^{(1)}(x)\left[nx j_{ml}(nx)\right]'}$$

$$b_{ml} = \frac{j_{ml}(nx)\left[x j_{ml}(x)\right]' - n^2 j_{ml}(x)\left[nx j_{ml}(nx)\right]'}{j_{ml}(nx)\left[x h_{ml}^{(1)}(x)\right]' - n^2 h_{ml}^{(1)}(x)\left[nx j_{ml}(nx)\right]'}$$

Here $n = \epsilon^2$ is the refractive index of the spheres; $x = kr_0$ is the size parameter; j_{ml}, h_{ml} are the spherical Bessel and Hankel of the first kind functions respectively.

In the case of $l \gg 1$ the calculation of the translational coefficients can be significantly simplified with the help of the so-called maximum term approximation [9].

$$
\begin{aligned}
A_l^{l'} &\cong -2l\,(-1)^{l+1}\sqrt{\frac{l+l'}{\pi\,(l'+1)\,(l-1)}}\times \\
&\quad \frac{l^l (l')^{l'}}{(l'+1)^{l'+1}\,(l-1)^{l-1}} h_{l+l'}^{(1)}(\eta x) \\
B_l^{l'} &\cong i\frac{x\,|i-j|}{ll'}A_l^{l'}
\end{aligned}
$$

Here η defined as $\eta = |r_0 + \delta r|/r_0 \geq 1$ is a dimensionless distance between the centers of the spheres.

References

[1] G. Kavoulakis and G. Baym, Phys. Rev. B **53**, 7227 (1996).

[2] O. Roslyak and J. Birman, arXiv:cond-mat/0703650, PRB to be published (2007).

[3] D. Frohlich, G. Dasbach, G. B. Hogersthal, M. Bayer, R. Kliebera, D. Sutera, and H. Stolzb, Solid State Communications **134**, 139 (2005).

[4] C. Ell, A. L. Ivanov, and H. Haug, Phys. Rev. B **57**, 9663 (1998).

[5] D. Snoke, Science **298**, 1368 (2002).

[6] J. Kasprzak, M. Richard, S. Kundermann, A. Baas, P. Jeambrun, J. Keeling, F. Marchetti, M. Szymanska, R. Andre, J. Staehli, et al., Nature **443**, 409 (2006).

[7] H. W. Xudong Fan, Scott Lacey, Optics Letters **24**, 771 (1999).

[8] X. Fan, S. Lacey, and H. Wang, Opt. Lett **24**, 771 (1999).

[9] H. Miyazaki and Y. Jimba, Phys. Rev. B **62**, 7976 (2000).

[10] C. Bohren and D. Huffman, *Absorption and scattering of light by small particles* (Wiley New York, 1983).

[11] S. Stein, Q. appl. Math **19**, 15 (1961).

[12] K. Fuller, Appl. Opt **30**, 4716 (1991).

[13] H. J. Carmichael, Phys. Rev. A **33**, 3262 (1986).

[14] Y. Hara, T. Mukaiyama, K. Takeda, and M. Kuwata-Gonokami, Physical Review Letters **94**, 203905 (2005).

[15] L. Deych and A. Roslyak, Physical Review E **73**, 36606 (2006).

[16] E. Peter, P. Senellart, D. Martrou, A. Lemaître, J. Hours, J. Gérard, and J. Bloch, Physical Review Letters **95**, 67401 (2005).

[17] A. J. Leggett, Rev. Mod. Phys. **73**, 307 (2001).
