# Supplementary material for: Caretta – A multiple protein structure alignment and feature extraction suite
Source: Comput Struct Biotechnol J. 2020 Apr 6;18:981–92. doi: 10.1016/j.csbj.2020.03.011 (PMC7186369; doi:10.1016/j.csbj.2020.03.011)
Supplement: Supplementary file 1 [file mmc1.zip › doc/jfigs.pdf]

Remark 3. We remark that; when the ratio h/λ tends to 0, the expression $\lambda L(r,s) = -(s-r)/(4\left(\frac{h}{\lambda}\right)^2 + (r-s)^2)$ tends to $1/(r-s)$ which is a singular function. This means that the expression $\lambda L(r,s)$ is not well behaved for the small values of h/λ. Consequently, for the solution to converge, the integrals of (10) and (11) must be evaluated with a large number of nodes. In our numerical applications (cf. section 5), we use 100 nodes to evaluate these integrals. With the smallest value of $h/\lambda = 0.02$, the convergence is good with $N = 20$.

Theorem 2. *For system (8), consensus can be achieved with $\|T_{\omega z}(s)\|_\infty < \gamma$ if there exist a symmetric positive definite matrix $P \in \mathcal{R}^{(n-1)\times(n-1)}$ and a scalar $\mu > 0$ satisfying*

$$\Gamma = \begin{bmatrix} -\bar{L}^T P - P\bar{L} + U_1^T U_1 + \mu \bar{E} & PU_1^T E_1 & PU_1^T \\ E_1^T U_1 P & -\mu I & 0 \\ U_1 P & 0 & -\gamma^2 I \end{bmatrix} < 0, \tag{10}$$

where $\bar{L} = U_1^T L U_1$ and $\bar{E} = U_1^T E_2^T E_2 U_1$.

Proof of Theorem 2. Proof follows straightforward from Lemma 3 and Theorem 1. However, it should be emphasized that all possible $\bar{L}_{\sigma(t)}$ should share a common Lyapunov function $V(\delta) = \delta^T(t)P\delta(t)$ (see the proof of Lemma 3 in Appendix A). □

1. The enumerate environment starts with an optional argument ‘1.’ so that the item counter will be suffixed by a period.
2. You can use ‘(a)’ for alphabetical counter and ’(i)’ for roman counter.
    a) Another level of list with alphabetical counter.
    b) One more item before we start another.
        (i) This item has roman numeral counter.
        (ii) Another one before we close the third level.
    c) Third item in second level.
3. All list items conclude with this step.

Step 1. This is the first step of the example list.
Step 2. Obviously this is the second step.
Step 3. The final step to wind up this example.

```
\includegraphics[width=3in,angle=45]{tiger.pdf}
```

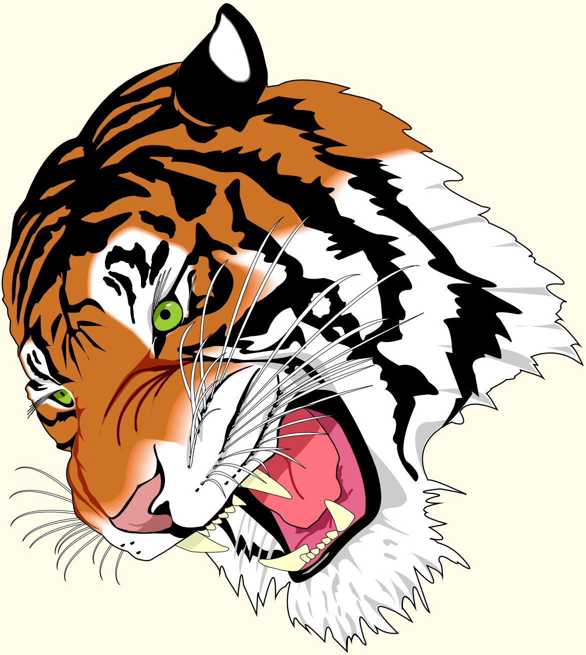

Fig. 1. More details on the usage of `\includegraphics` can be found in the grfguide.ps of the LaTeX documentation.

[1] Knuth, D.E., *TeX: The Program*, Computers & Typesetting; B., 1995, Addisson-Wesley Publishing Co., Inc., New York.
