# Supplementary figures and images for: Caretta – A multiple protein structure alignment and feature extraction suite
Source: Comput Struct Biotechnol J. 2020 Apr 6;18:981–92. doi: 10.1016/j.csbj.2020.03.011 (PMC7186369; doi:10.1016/j.csbj.2020.03.011)

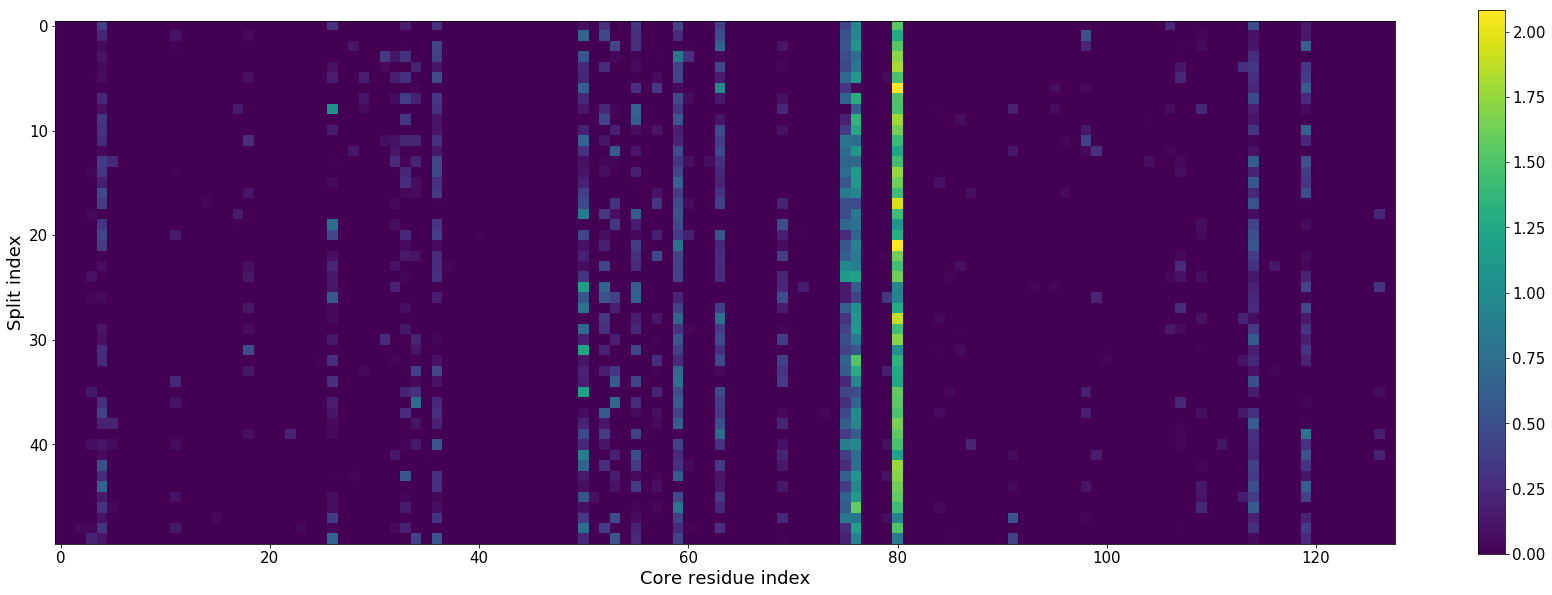

Supplement: Supplementary file 1 [file mmc1.zip › images/important_residues.png]

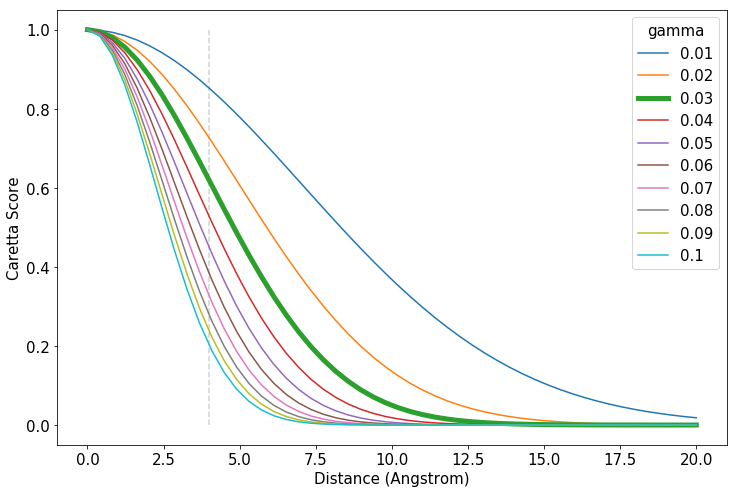

Supplement: Supplementary file 1 [file mmc1.zip › images/plot_gamma.png]

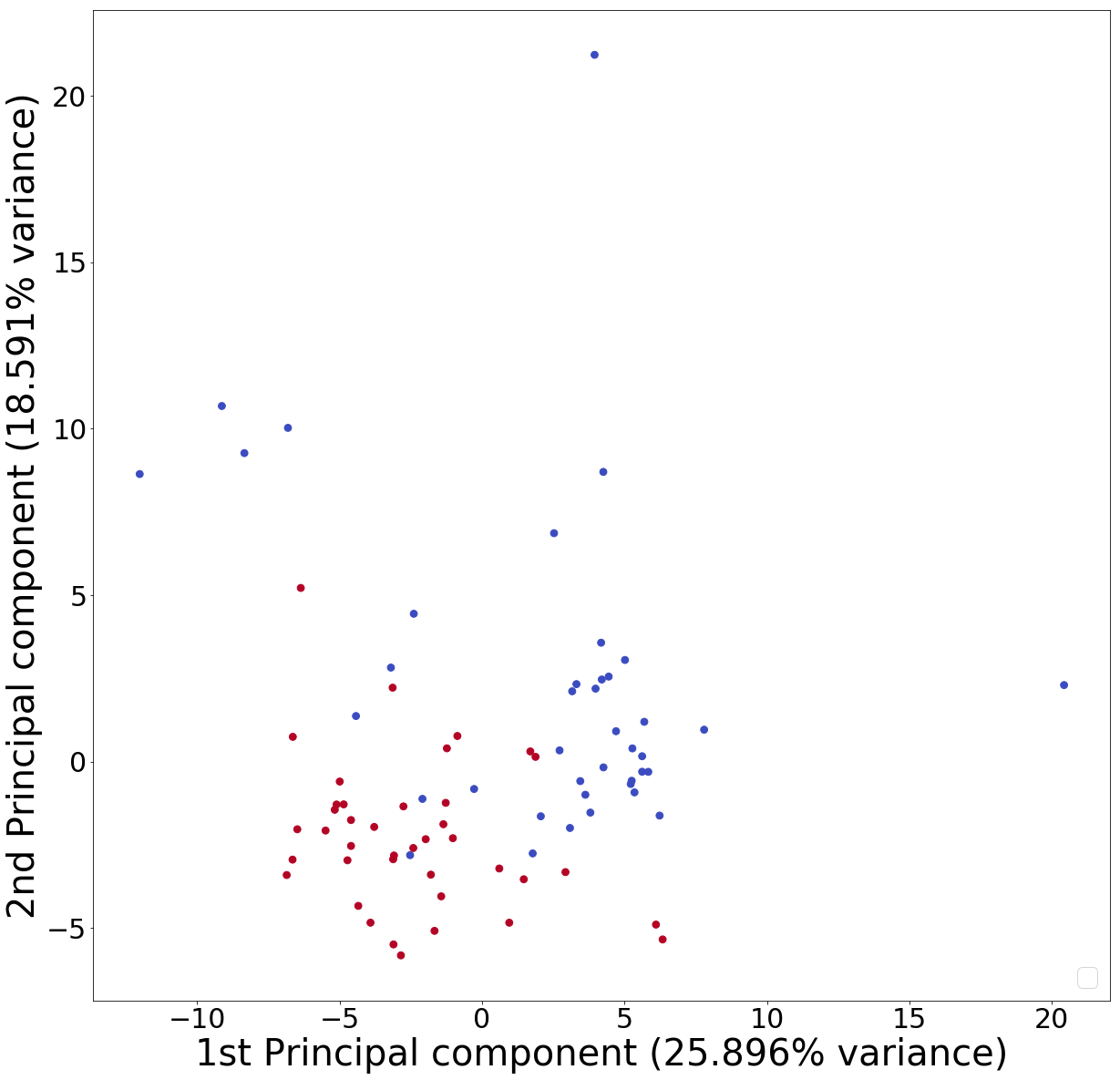

Supplement: Supplementary file 1 [file mmc1.zip › images/important_features.png]
